# A Darwinian model for the evolution of drug resistance to long-acting PrEP during an early HIV infection

**DOI:** 10.1371/journal.pone.0355833

**Published:** 2026-08-20

**Authors:** Katharine Gurski, Yeona Kang, Yanping Ma

**Affiliations:** 1 Department of Mathematics, Howard University, Washington, District of Columbia, United States of America; 2 Department of Mathematics, Statistics and Data Science, Loyola Marymount University, Los Angeles, California, United States of America; Icahn School of Medicine at Mount Sinai Department of Pharmacological Sciences, UNITED STATES OF AMERICA

## Abstract

Predicting the evolution of drug resistance remains a central challenge in HIV prevention and treatment. Evolutionary trade-offs, i.e., the fitness costs of resistance, shift as drug-selective pressure changes, a complexity amplified by fluctuating drug concentrations during long-acting prophylaxis. We develop a Darwinian evolutionary game theory model of early HIV infection under bimonthly pre-exposure prophylaxis (PrEP) with long-acting cabotegravir (CAB-LA). This work introduces a mathematical framework that couples within-host viral dynamics with adaptive evolutionary processes in a time-dependent environment to study resistance evolution under long-acting PrEP. The model incorporates the dual transmission pathways of HIV: free virion spread and direct cell-to-cell transfer through virological synapses. We quantify how resistance mutations alter viral fitness across these modes. We formulate a deterministic within-host model that explicitly incorporates the dual transmission pathways and introduce continuous resistance traits governing infectivity and drug susceptibility for each pathway. T-cell parameter estimates are informed by data assimilation using acute-stage HIV infection data. CAB-LA is modeled with time-periodic pharmacokinetics–pharmacodynamics (PK/PD), yielding dynamic fitness seascapes rather than static fitness landscapes. Time-varying drug pressure introduces a trade-off between drug resistance and infectivity, driving competition among strains that favor different transmission strategies. Our results show that fluctuating drug concentrations reshape evolutionary outcomes, generating dynamic strain competition and altering the effectiveness of long-acting PrEP in blocking infection. The model reveals threshold behavior in drug robustness to mutation, identifies conditions leading to viral control, viral escape, and delayed seroconversion, and demonstrates that resistance evolution depends on both transmission pathway and initial trait configuration. These findings provide a mechanistic explanation for delayed HIV detectability observed in CAB-LA clinical trials.

## 1 Introduction

The emergence of drug resistance in HIV remains a critical challenge for both treatment and prevention strategies, including long-acting pre-exposure prophylaxis (PrEP) regimens. Sustained drug pressure from these therapies creates an evolutionary trade-off between resistance and infectivity. While early mutations in HIV were once thought to be largely neutral, i.e., having little impact on fitness or reducing infectivity enough to prevent their spread, recent analyses of global mutational fitness landscapes and sequencing data of the *env* gene from individuals with acute infection suggest otherwise [1,2]. Approximately 5% of early-stage mutations confer a measurable fitness advantage [1,2]. These observations indicate that the neutral-mutation assumption is overly restrictive; drawing fitness effects from a log-normal distribution reveals a broader spectrum of mutations beneficial to viral growth, underscoring that selection acts even at the onset of infection. [3].

HIV’s high mutation rate, estimated at $1.1\times 10^{-5}$ and $5.8\times 10^{-3}$ mutations per nucleotide per replication [4–7], further amplifies this potential. Given the approximately 9,200 nucleotides in the HIV genome and a 24-hour replication cycle, this implies that the expected waiting time for a specific point mutation is approximately 10.9 days. In this study, we focus on the acute phase of infection to isolate these initial, rapid evolutionary and transmission processes.

In the context of bimonthly long-acting PrEP injections, viral evolution unfolds under fluctuating drug concentrations, which reshape selective pressures and may accelerate adaptation. Capturing these dynamics requires a framework that explicitly models fitness trade-offs. Evolutionary game theory (EGT) provides such a framework by quantifying frequency-dependent selection and strategic interactions within biological systems. Since the foundational work of Maynard Smith and Price [8], EGT has been applied to study adaptation under selective pressure. Here, the time-varying concentration of long-acting PrEP transforms the fitness landscape into a dynamic fitness seascape [9]. Following [10], we treat adaptation as a non-equilibrium response, providing a mechanistic basis for understanding resistance emergence under intermittent drug pressure. Biologically, this perspective highlights that resistance-conferring traits may be advantageous at low drug concentrations but costly when concentrations are high, producing time-dependent fitness trade-offs.

Our approach builds on the EGT model of Cushing et al. [11] in which the mean evolutionary dynamics follows Lande’s (Fisher’s canonical) equation of evolution [12].

The present work makes three contributions that distinguish it from prior HIV modeling efforts. First, we explicitly couple within-host viral dynamics with time-dependent pharmacokinetics and pharmacodynamics (PK/PD) of CAB-LA using an evolutionary game theory (EGT) framework. While prior within-host HIV models have incorporated PK/PD or resistance, none have linked time-varying drug concentrations to a fitness seascape that continuously reshapes evolutionary selection pressures. Second, we separately model the two transmission pathways, free virion spread and cell-to-cell transfer via virological synapses, as distinct evolutionary strategies with independent resistance traits ($u_{k}$ and $u_{w}$). Existing resistance models treat infection as a single route; our dual-pathway structure reveals that CAB-LA exerts asymmetric selective pressure on the two pathways, with important consequences for resistance emergence and delayed seroconversion. Third, we provide a mechanistic, within-host explanation for the delayed seroconversion reported in the clinical trials HPTN 083 and 084 [13,14], grounding the clinical observation in the trade-off dynamics between drug resistance and infectivity under evolving phenotypic traits. To our knowledge, no prior model has addressed this specific question at the within-host level.

Using this Darwinian framework, we investigate three central questions:

- Under what conditions can a bimonthly regimen of long-acting PrEP maintain viral control despite evolving resistance?
- How strongly does time-varying drug concentration influence viral escape by resistant strains?
- Does the interplay of PrEP and resistance evolution delay the detection of infection via standard HIV testing?

This work represents an initial step toward optimizing long-acting PrEP protocols to suppress resistance while maintaining viral control, thus linking evolutionary dynamics to the design of clinical intervention.

To address these questions, the remainder of the paper proceeds as follows. In Section 2, we discuss the use of EGT in disease modeling. Section 3 introduces the background on a new long-acting PrEP medication and the potential for drug resistance. In Section 4, we describe the mechanism of HIV replication and how integrase inhibitors function. Section 5 presents the model for the time-varying drug concentration. In Section 6, we explain the differences and similarities between the cell-to-cell and free virion transfer mechanisms. The cellular HIV model with PrEP is developed in Section 7 with measures of drug efficacy needed to maintain prevention of HIV infection after exposure. In Section 8, we apply EGT to our cellular model to understand how an initial exposure to a resistant strain will evolve under CAB-LA. Section 9 provides a mechanism for delay in HIV detection using standard testing. Finally, Section 10 outlines our conclusions and future directions.

## 2 EGT and fitness in disease models

In a Darwinian framework, HIV mutations are expected to evolve in ways that maximize viral fitness. Early interpretations of fitness assumed pathogens would evolve to minimize host mortality [15], but mathematical models later demonstrated that transmission success can sustain virulence above minimal levels [16–18]. Changes in fitness as a function of genotype are often visualized as a fitness landscape [19]. Yet, because fitness varies with time and environment, a static landscape provides only a snapshot. EGT extends this view by modeling fitness dynamically, leading to the concept of an adaptive landscape, where selection unfolds in a continually shifting environment.

EGT has been widely applied to disease modeling, to clarify host–pathogen interactions, immune evasion, and the evolution of drug resistance [20]. For HIV, EGT has provided insight into viral adaptation of an HIV infection under antiretroviral treatments [15], while more recent work combines EGT with quantum game theory to model within-host infectivity and replication [21,22]. Beyond virology, EGT has gained traction in oncology, where heterogeneous tumor cell populations compete under selective pressure from therapy [23–26]. Here, fitness is shaped by ecological and evolutionary dynamics rather than health or longevity, as emphasized by Gluckman et al. [27].

Building on this principle, Staňková et al. [28] framed cancer therapy as a Stackelberg evolutionary game, with physicians guiding tumor evolution through dynamic treatment strategies, while Zhang et al. [29] demonstrated that evolution-informed approaches can delay resistance in prostate cancer. Together, these examples illustrate the utility of EGT in managing resistance in complex biological systems.

**Fig 1:**
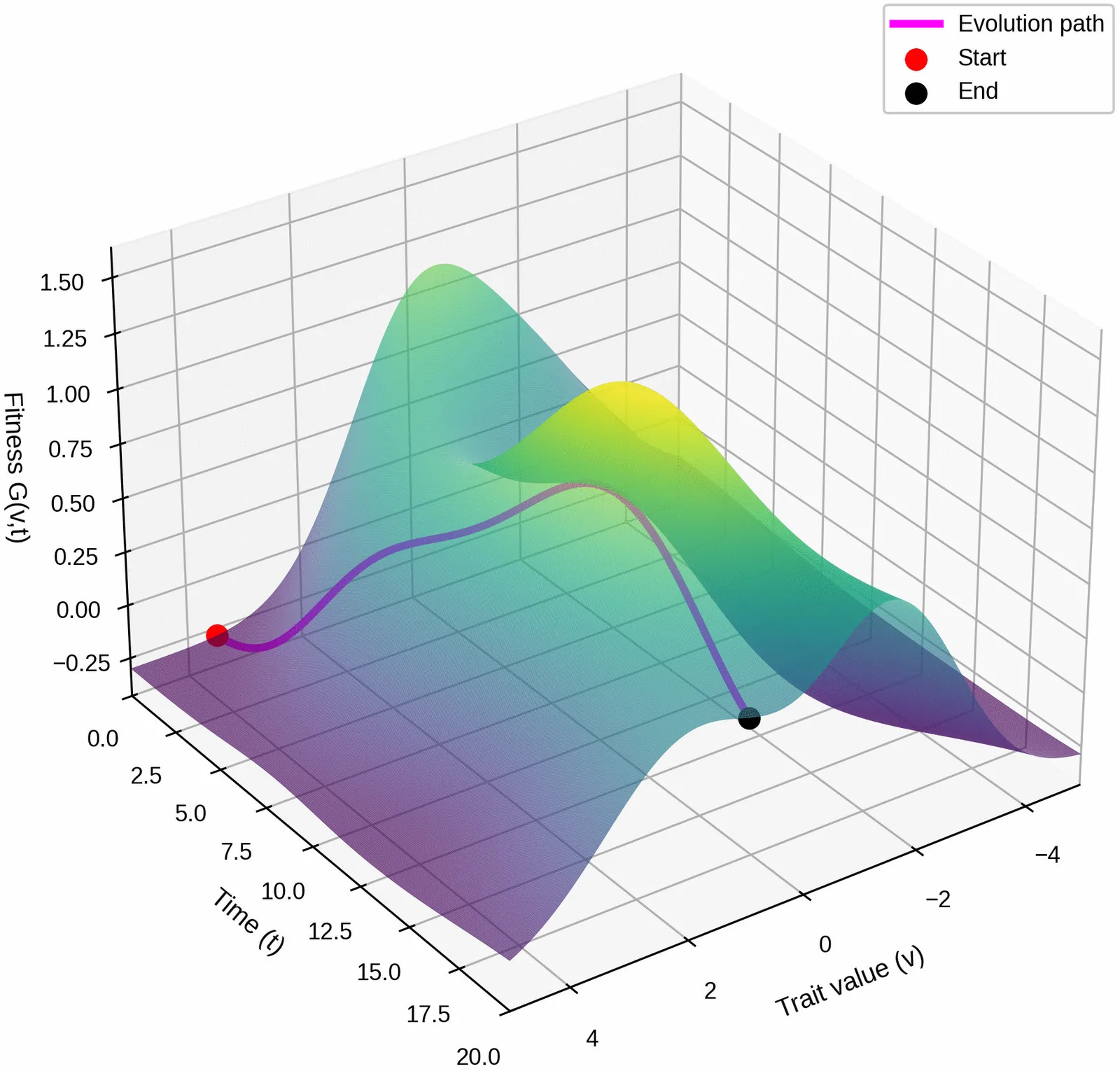
Adaptive Trajectory on a Dynamic Fitness Landscape. The surface illustrates how fitness peaks and valleys change over time, guiding the evolutionary path from start (red) to end (black).

In oncology, these eco-evolutionary treatment dynamics are often summarized as adaptive landscapes where fitness depends on evolving drug-resistant traits [30]. However, HIV under long-acting PrEP poses a distinct challenge. Long-acting PrEP is administered as a single injection every eight weeks, producing a time-dependent decline in drug concentration. This requires a shift from adaptive landscapes to fitness seascapes [9], which are collections of trait-specific, dose-dependent fitness curves that explicitly incorporate temporal variation. Fitness seascapes capture the interaction between viral evolution, drug resistance, and pharmacokinetics by linking resistance trade-offs with fluctuating selective pressures. Fig 1 illustrates this conceptual shift, showing how evolutionary trajectories unfold on a surface that is not static but continuously reshaped by both ecological and pharmacological forces.

King et al. [31] recently employed a fitness seascape framework to study bacterial resistance, measuring dose-dependent adaptation of E. coli to cefotaxime. They observed significant growth penalties associated with resistance in a fluctuating drug environment, highlighting the role of dynamic selection. Analogously, bimonthly long-acting PrEP generates a gradually declining drug profile between injections. In our work, we integrate both injection timing and drug decay kinetics to construct a within-host fitness seascape for HIV under long-acting PrEP, enabling us to quantify the dose-dependent evolution of resistant strains during initial exposure and acute infection.

## 3 Background: Drug resistance of HIV to long-acting PrEP

In December 2021, the U.S. Food and Drug Administration (FDA) approved Apretude (cabotegravir long-acting, CAB-LA) for HIV prevention in adults and adolescents [32]. CAB-LA, an integrase strand transfer inhibitor (INSTI), is administered as two injections given 2 weeks apart, followed by maintenance injections every eight weeks [33]. The ECLAIR trial demonstrated its long half-life, with detectable drug levels persisting for 52 weeks after the final injection in 14% of participants [34]. This prolonged pharmacokinetic profile enables infrequent dosing but also produces a long “pharmacological tail,” during which drug concentrations may be too low for protection yet sufficient to select for resistance if PrEP is discontinued [35].

CAB-LA also alters the dynamics of HIV seroconversion. In clinical trials, CAB-LA exposure delayed viral detectability and antibody expression, leading to postponed diagnosis and, in some cases, inadvertent dosing after infection had occurred [36,37]. Such delays have been linked to the emergence of INSTI resistance [38]. Two large trials, HPTN 083 and HPTN 084, confirmed that CAB-LA outperformed daily oral tenofovir disoproxil fumarate/emtricitabine (TDF/FTC) in men who have sex with men, transgender women, and women in sub-Saharan Africa [13,14]. However, both trials reported delayed HIV diagnosis and, in some cases, INSTI-resistant infections.

Evidence from macaque studies supports this concern: CAB-LA exposure prior to seroconversion can select for resistant SHIV variants [39]. Similarly, insufficient viral suppression during antiretroviral therapy (ART) enables resistant HIV mutants to arise within days [40]. These risks informed FDA recommendations for HIV testing before each CAB-LA injection [32]. While resistance in PrEP users has been rare in randomized trials, CAB is also used in ART regimens, and resistance could compromise treatment efficacy if it emerges [41].

At the molecular level, resistance to integrase inhibitors often follows specific mutational pathways, notably Y143 and Q148, which maintain viral fitness while conferring resistance [41]. Transmission studies further show that the spread of resistant HIV depends on both fitness costs and transmission bottlenecks: low-cost mutations may propagate, while high-cost mutations often revert to wild type or persist at undetectable levels [42]. A bimonthly injection regimen reduces adherence issues that contribute to resistance during oral regimens, but viral fitness and transmission dynamics remain central to resistance risk.

Mathematical modeling has been used to study drug resistance and HIV transmission but has rarely addressed long-acting PrEP specifically. Cox et al. [43] applied the agent-based EMOD-HIV model to simulate oral PrEP expansion in Kenya, while Smith et al. [44] used the HIV Synthesis model to compare CAB-LA with oral PrEP under heterosexual transmission scenarios. Both approaches incorporated drug resistance only indirectly, without explicitly modeling mutation processes. At the within-host level, numerous models have examined HIV cellular dynamics [45–49], but most exclude resistance. In earlier work, we developed a mechanistic ODE model of HIV and SHIV infection under CAB-LA, incorporating pharmacokinetics and pharmacodynamics [50]. That model identified viral infectivity and CAB-LA efficacy against resistant strains as key determinants of resistance emergence, but it did not explicitly couple infectivity with drug efficacy. In the present study, we address this limitation by coupling trait-dependent infectivity with drug-modified progression rates within an EGT framework.

## 4 HIV replication and integrase inhibitors

**Fig 2:**
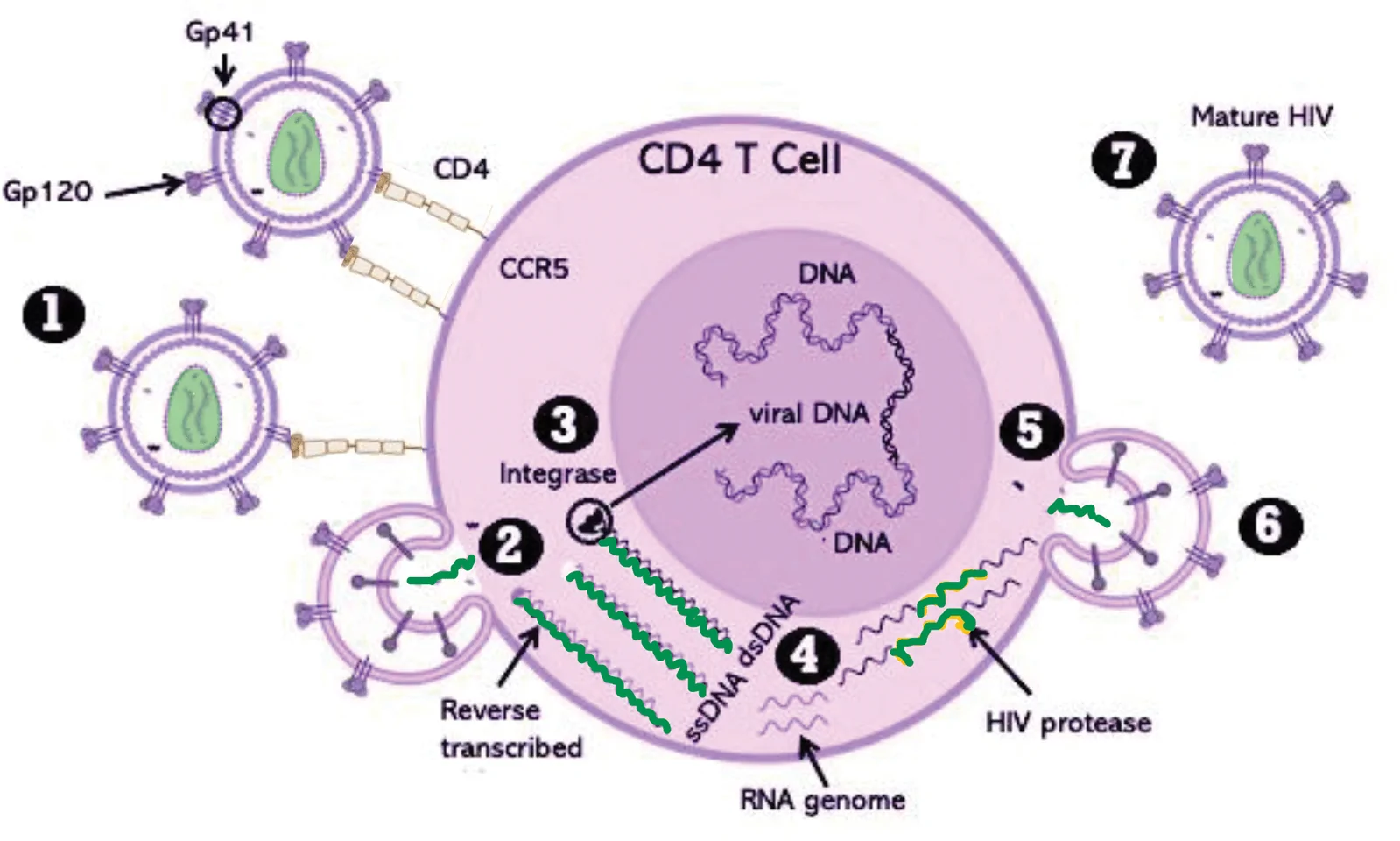
Schematic of the HIV life cycle. Stage numbers denote the targets of specific antiretroviral drugs. CAB-LA is an integrase inhibitor that works at stage 3 by blocking integration of viral DNA into the host genome.

HIV targets CD4^+^ T-cells, a type of white blood cell critical for coordinating adaptive immune defense. The HIV life cycle [51] consists of a series of steps by which the virus infects cells, replicates, and spreads (Fig 2). First, HIV attaches to CD4^+^ T-cells via its envelope glycoprotein GP120 (stage 1), fuses with the cell membrane, and enters the host cell. Once inside, the viral RNA is reverse-transcribed into DNA (stage 2), which is then integrated into the host genome by the viral integrase enzyme (stage 3). The integrated DNA (provirus) is transcribed into RNA and translated into viral proteins (stage 4). These proteins and RNA assemble into new virions (stage 5), which bud from the host cell (stage 6) and are released into circulation to infect additional cells (stage 7).

After integration, HIV is subject to strong selective pressure from innate and adaptive immunity, driving frequent mutations [40]. As an RNA virus, HIV has inherently low replication fidelity: intracellular assays estimate a mutation rate of roughly 10^−5^ per nucleotide per replication cycle, similar to other retroviruses [40,52–54]. Throughout this paper, HIV refers primarily to HIV-1, which is globally prevalent, whereas HIV-2 — less transmissible and less likely to progress to AIDS — is largely confined to West Africa.

PrEP formulations act at different stages of this replication cycle. Integrase inhibitors such as CAB-LA block the integration step (stage 3), preventing viral DNA from inserting into the host genome. By contrast, nucleoside and non-nucleoside reverse transcriptase inhibitors (NRTIs, NNRTIs) act earlier, during reverse transcription (stage 2). These compounds mimic DNA building blocks and are incorporated into the nascent viral DNA chain by reverse transcriptase, causing premature termination. Thus, while both integrase inhibitor-based and NRTI-based PrEP regimens prevent infection, they operate through distinct molecular mechanisms. In our modeling framework below, we represent CAB-LA specifically as an inhibitor of the integration step, consistent with its mechanism of action at stage 3 of the life cycle.

## 5 Model for CAB-LA drug effectiveness

We model the pharmacokinetics and pharmacodynamics (PK–PD) of CAB-LA using a one-compartment framework. Drug effectiveness, $F_{eff}(t)$, is described by the median-effect model, derived from mass action principles and commonly expressed through the Hill equation [55]:


$$\begin{array}{c} F_{eff}(t)=1-\frac{IC_{50}^{n}}{IC_{50}^{n}+C(t{)}^{n}}, \end{array}$$ (1)

where *C*(*t*) is the drug concentration at time *t*, *IC*_50_ is the drug concentration that produces 50% of the inhibitory effect, and *n* is the Hill coefficient that governs the slope of the dose-response curve. Integrase inhibitors such as CAB-LA typically exhibit n≈1, consistent with non-cooperative binding [56].

For HIV, the experimental studies usually report the concentration required for 90% inhibition, rather than *IC*_50_. The two are related by the conversion formula:


$$\begin{array}{c} IC_{X}={\left(\frac{X}{100-X}\right)}^{1/n}IC_{50}. \end{array}$$ (2)

Thus, when *n* = 1, $IC_{50}=IC_{90}/9$.

**Fig 3:**
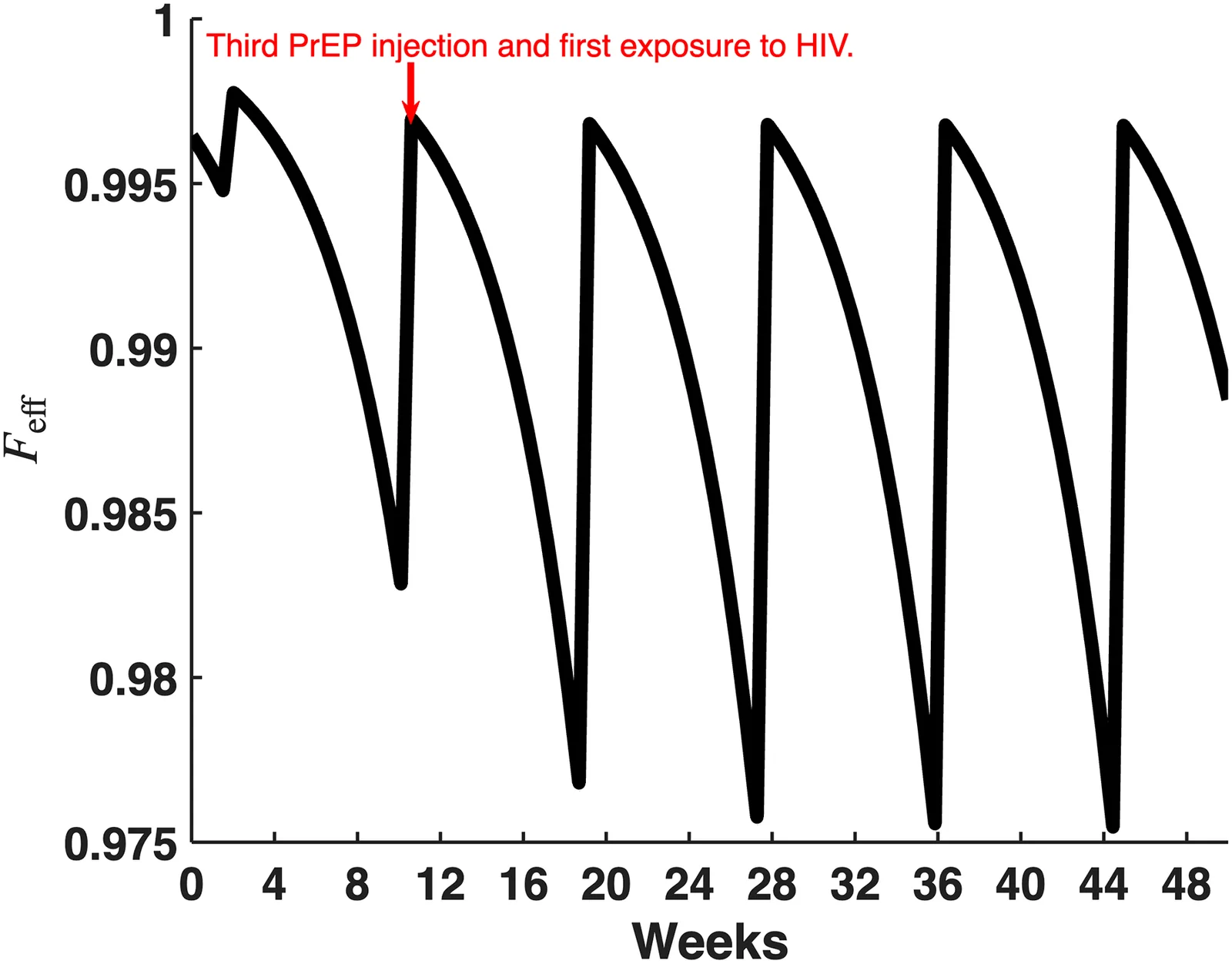
Drug effectiveness. Time course of the fraction of infection events affected by the drug, $F_{eff}(t)$, following the first injection in humans. The dosing schedule follows the WHO guideline [33] (July 2022), which recommends two initial injections 2 weeks apart, followed by subsequent injections every 2 months. The red arrow indicates the timing of the third injection. In our later analyses and simulations, HIV exposure is assumed to occur at this third injection.

In previous work [50], we fitted an exponential decay model to CAB-LA plasma concentrations in humans following a single injection, based on the data of Shaik et al. [57]. The fitted dose–response curve for the fraction of infection events blocked by the drug is:


$$\begin{array}{c} F_{eff}(t)=1-\frac{0.018}{0.018+5.04e^{-0.0363t}}, \end{array}$$ (3)

where the decay constant $0.0363\;{day}^{-1}$ corresponds to a half-life *h* such that ln(2)/h=0.0363. The inhibitory effect $F_{eff}(t)$ represents the fraction of viral integration events prevented by CAB-LA. As shown in Fig 3, effectiveness remains high, between 98% and 99.5%, for up to 8 weeks post-injection.

In our simulations, we assume that individuals initiate HIV exposure immediately after their third injection. This represents a conservative scenario in which drug concentrations are near peak effectiveness at baseline, allowing us to evaluate PrEP protection both at maximum coverage and during the natural decline of plasma concentrations.

## 6 Free virion transfer and cell-to-cell transfer

**Fig 4:**
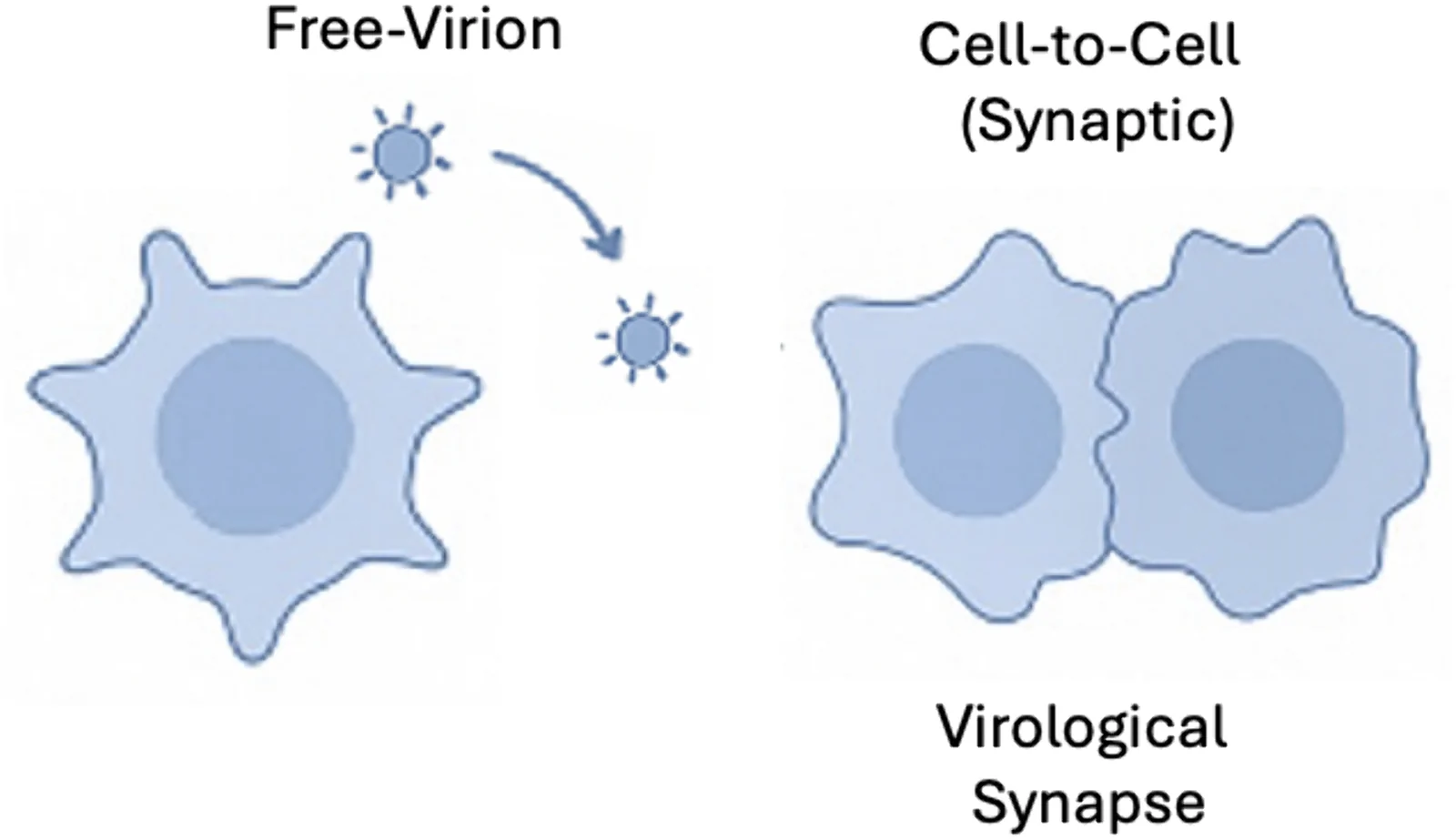
Free virion and cell-to-cell transfer. In free virion transfer, virions are released into the extracellular environment and infect susceptible T-cells through extracellular spread. In cell-to-cell transfer, a virological synapse forms, enabling direct passage of viral particles from an infected cell to a target cell.

There are two primary mechanisms of HIV transmission: free virion spread and cell-to-cell transfer [58,59]. Free virions, the extracellular form of the virus, are essential for spreading between distant cells and for transmission between hosts. To establish infection, free virions must bind to cell-surface receptors on CD4^+^ T-cells, thereby initiating the viral life cycle [60] (See Fig 4). However, free virions are also exposed to immune defenses, including antibody neutralization, antibody-dependent cellular cytotoxicity, and phagocytosis [61]. By contrast, cell-to-cell transmission occurs through a specialized junction known as the virological synapse. Quantitative 3D live microscopy has directly observed the budding of HIV particles into the synaptic cleft and their subsequent fusion with adjacent T-cells [62]. Multiple studies have shown that this pathway enables more rapid viral spread than free virion transmission [63–66].

Cell-to-cell transfer is advantageous for the virus in two ways: (i) receptor recruitment at the contact site makes entry more efficient [64,67,68], and (ii) direct transfer provides partial shielding from immune responses [69]. Indeed, as many as $10^{2}-10^{3}$ virions can be transmitted in a single synaptic event [62,64]. Fitting a mathematical model to *in vitro* data, Komarova et al. [70] concluded that both free virion and cell-to-cell pathways contribute comparably to overall viral growth.

Sigal et al. [69] demonstrated *in vitro* that the high efficiency of cell-to-cell transmission can reduce the effectiveness of intracellular antiretroviral agents, including reverse transcriptase inhibitors. However, Agosto et al. [71,72] showed that most NNRTIs (acting at stage 2), protease inhibitors (stage 4), and entry inhibitors (stage 1) retain substantial efficacy even when infection occurs through cell-to-cell transfer. To date, whether integrase strand transfer inhibitors (INSTIs), including CAB-LA, effectively suppress cell-to-cell infection has not been directly tested experimentally. Nevertheless, the findings of Agosto et al. [71,72] provide indirect support for the possibility that CAB-LA may retain at least partial efficacy along this transmission route. Because this remains an unresolved empirical question, our model explores a range of scenarios reflecting the extent to which CAB-LA suppresses cell-to-cell transmission across different levels of the viral resistance trait.

Modeling both free virion and cell-to-cell transmission is well established in within-host HIV models. For instance, Spouge et al. [73] introduced a predator–prey framework to study HIV spread in tissue cultures. Building on this tradition, our work extends the within-host ODE model of Graw and Perelson [47], providing a deterministic framework for quantifying HIV dynamics under PrEP. With this foundation, we introduce a new model that explicitly represents this dual-pathway structure through separate compartments for infection initiated by free virions and by cell-to-cell transfer to assess the effectiveness of long-acting PrEP in suppressing viral spread.

## 7 Cellular model of HIV prevention with PrEP

We extend the Graw–Perelson model [47] by dividing infected T-cells into two categories: early infection (*E*) and late infection (*I*). In our framework, T-cells in stages 1 or 2 of the HIV life cycle (see Fig 2) are classified as “early” (*E*), meaning they cannot yet produce new viral proteins. Once the viral DNA integrates into the host genome (stage 3), infected T-cells move into the “late” (*I*) category and gain the ability to produce new virions. Because HIV replication requires integration of viral DNA into the host cell genome, an integrase inhibitor like CAB-LA blocks the transition from *E* to *I*. Consequently, our model captures the effect of CAB-LA precisely at this critical step. To emphasize the early-stage effects of PrEP, we restrict our analysis to the acute phase of HIV infection, excluding chronic stages and development of latent reservoirs.

**Fig 5:**
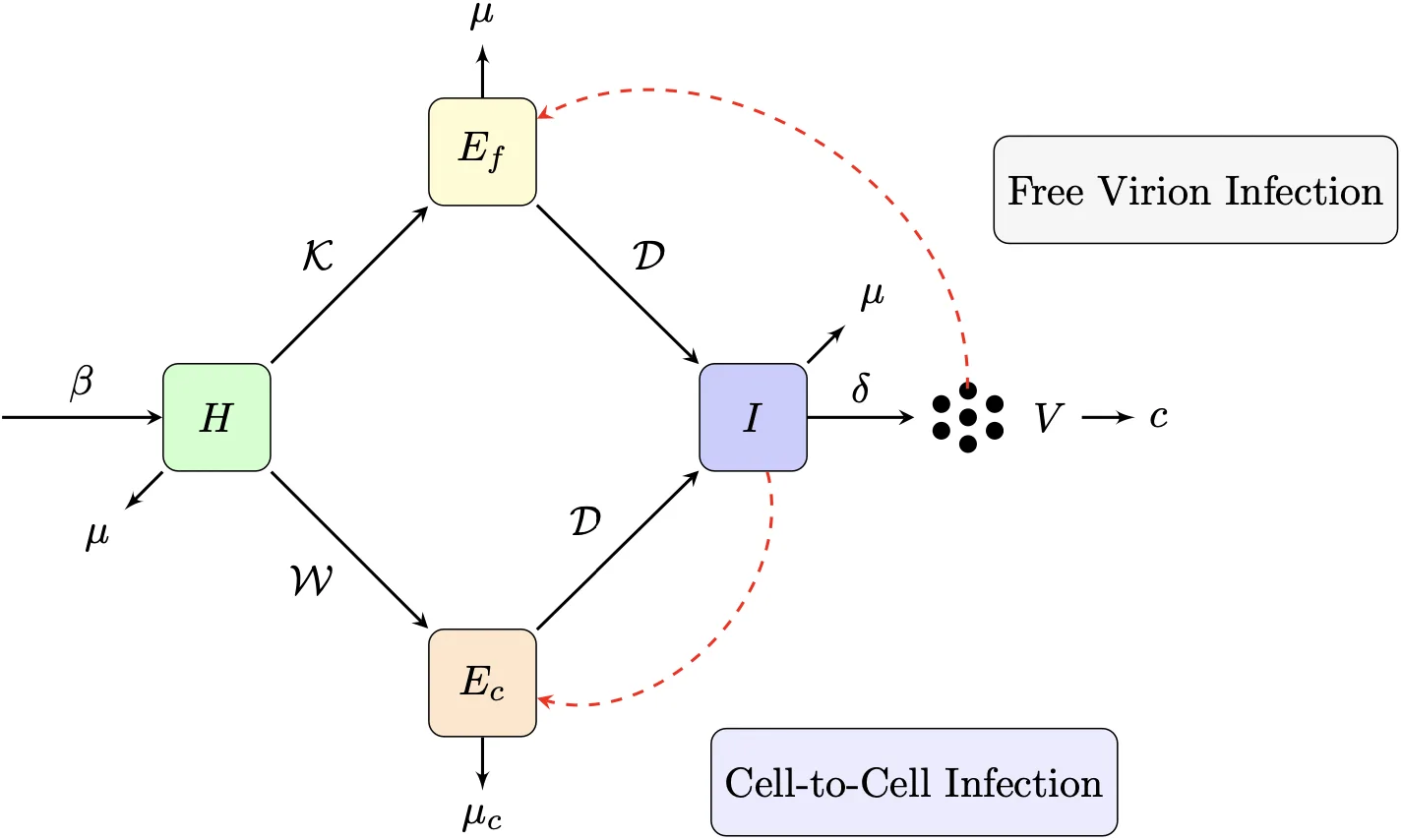
Model with free virion and cell-to-cell infection. Solid black arrows denote transitions between population stages while red dashed arrows represent infection routes into the early infected T-cells.Healthy T-cells are denoted by *H*, free virions by *V*, early infected T-cells by free virion transfer by $E_{f}$, early infected T-cells by cell-to-cell transfer by $E_{c}$, and productively infected T-cells by *I*. CAB-LA acts by reducing progression from early infection to productive infection.

We now define the other state variables. Healthy T-cells are denoted by *H*, and free virions by *V*. Early infected T-cells are further divided into two classes: those infected by free virions ($E_{f}$) and those infected via cell-to-cell transmission ($E_{c}$). Although T-cells infected by cell-to-cell transfer are thought to have a greater propensity for latency [74], we do not explicitly include latent compartments in this acute-stage HIV model (Fig 5). Early infection T-cells progress to productive infection at a rate ϕ. Since the HIV life cycle is approximately 24 hours and integration occurs roughly 9 hours after infection, we set $\phi =24/15\;{days}^{-1}$ [75,76]. Productively infected cells eventually burst, releasing virions into the plasma pool *V*. A summary of state variables is provided in Table 1.

**Table 1:** State variables and their initial conditions.

Variables	Definition (Concentrations of)	Initial conditions
*H*	Healthy T-Cells	10^3^ cells/μL
$E_{f}$	Early Infection T-cells — Free virion	0 cells/μL
$E_{c}$	Early Infection T-cells — cell-to-cell	0 cells/μL
*I*	Productively Infected T-Cells	10^−3^ cells/μL
*V*	Free Virions	100 virions/μL

Healthy CD4^+^ T-cells are recruited at the rate β and die at their natural death rate μ. They become infected by free virions at rate 𝒦 and via cell-to-cell transmission at rate 𝒲. Cells in $E_{f}$ die at rate μ, while those in $E_{c}$ die at the higher rate $\mu_{c}$. Productively infected cells (*I*) die both from natural loss (μ) and virus-induced death (δ). The baseline transition rate from *E* to *I* is ϕ, but under CAB-LA treatment, this rate is reduced by drug action. Specifically, the effective progression rate is


$$\begin{array}{c} 𝒟(t)=\left(1-F_{eff}(t)\right)\phi , \end{array}$$ (4)

where $F_{eff}(t)$ is the time-varying drug efficacy illustrated in Fig 3.

Each virus-induced death of a productively infected T-cell releases *N* virions, contributing at a rate δIN to the viral load. Free virions are cleared from plasma at rate *c*. While clearance rates vary during infection due to immune response dynamics, we assume constant values for simplicity, consistent with our acute-stage focus. Parameters and definitions are summarized in Table 2. The HIV infection system including both free virus and cell-to-cell transfer is given in the following system.


$$\begin{array}{cc} \frac{dH}{dt} & =\beta -\left[k_{m}V+\omega_{m}I+\mu \right]H,\\ \frac{dE_{f}}{dt} & =k_{m}VH-\left[𝒟(t)+\mu \right]E_{f},\\ \frac{dE_{c}}{dt} & =\omega_{m}IH-\left[𝒟(t)+\mu_{c}\right]E_{c},\\ \frac{dI}{dt} & =𝒟(t)\left[E_{f}+E_{c}\right]-\left[\delta +\mu \right]I,\\ \frac{dV}{dt} & =N\delta I-cV. \end{array}$$ (5)

**Table 2:** Definition of parameters and their values. Values from literature are listed with a citation under Source; otherwise, the value was calculated or fitted as noted.

Parameter	Definition	Value (Units)	Source
β	Recruitment rate of healthy T-cells	60 cells per day $\cdot \mu L^{-1}$	[77,78]
*c*	Free virion clearance rate	25 day^-1^	[77,79]
δ	Bursting rate of infected T-cells	1 day^-1^	[80]
μ	Natural death rate T-cells	0.05 day^-1^	[77,81]
$\mu_{c}$	Death rate of early infection due to cell-to-cell	0.25 day^-1^	[74]
*N*	Virions released	100 per infected cell	[80]
ϕ	Progression rate from early to productive (stage 3)	24/15 day^-1^	Calculated
$k_{m}$	Maximum (free virion) infection rate	$2.5\times 10^{-4}$ per virions $\cdot \mu L^{-1}$	[81]
$\omega_{m}$	Maximum (cell-to-cell) infection rate	$2.93\times 10^{-3}$ per infected cell $\cdot \mu L^{-1}$	Fitted

For the model in this section, $𝒦\equiv k_{m}$ and $𝒲\equiv \omega_{m}$, as in Table 2.

Reported estimates for $\omega_{m}$ vary widely, from $0.1k_{m}$ [45] to $100k_{m}$ [47]. To determine an appropriate value for $\omega_{m}$, we used the data assimilation technique known as nudging (or Newtonian relaxation). Nudging “relaxes” the dynamical model toward observed data by adding a linear term proportional to the difference between the model state and the observations [82]. In our case, the model was nudged toward viral load data from the RV217 cohort [83], which captured 155 acute HIV-1 infections across four countries.

**Fig 6:**
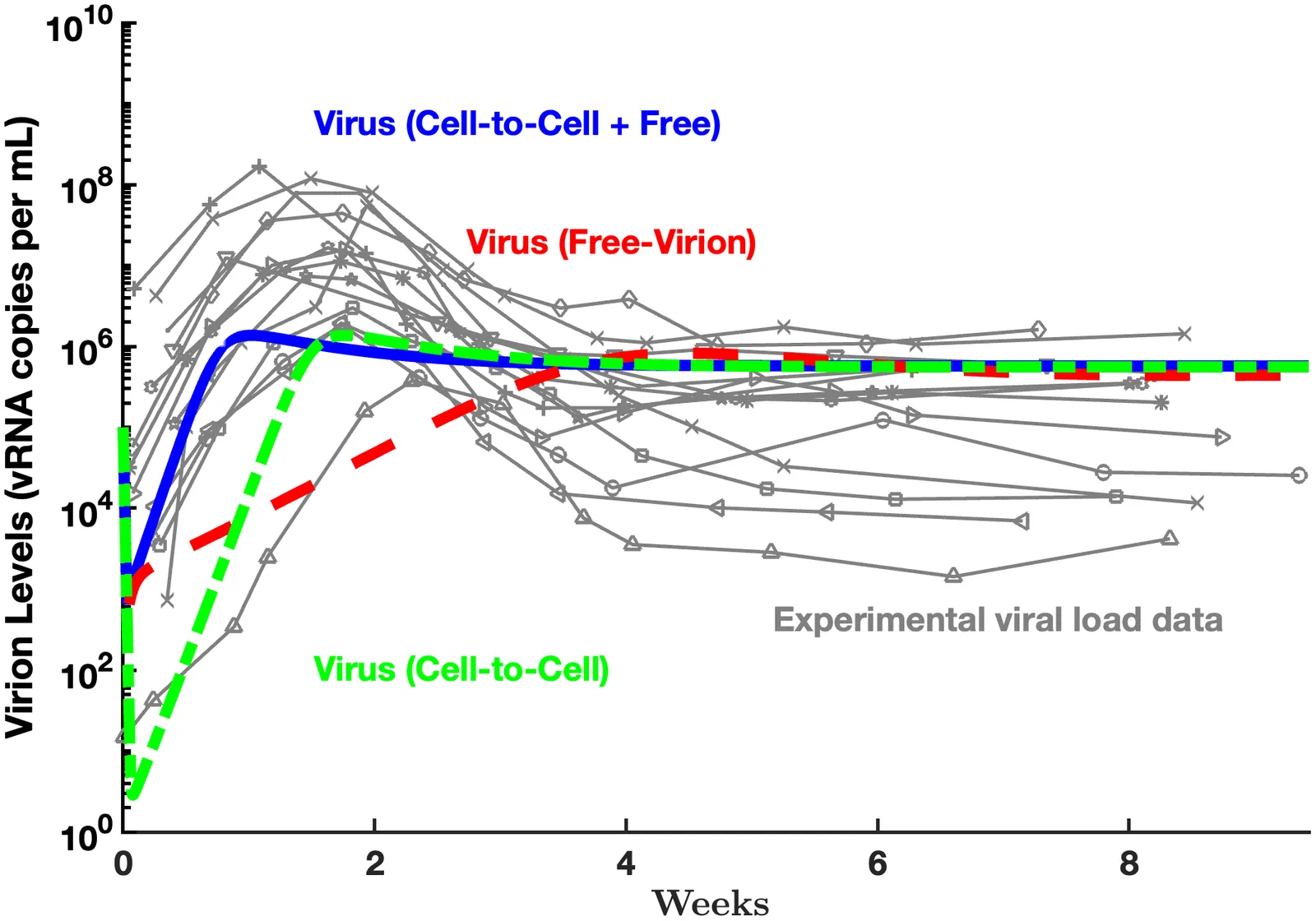
Viral load during acute HIV infection. Light gray points represent RV217 cohort data [83]. Red dashed curve: free virion transmission only. Green dash-dot: cell-to-cell transmission only. Blue solid: both pathways.The cell-to-cell transmission rate $\omega_{m}$ is fitted so that the full model captures the timing of peak viremia in the acute-stage viral-load data.

We first smoothed viral load data from 46 individuals [84] and used the resulting mean viral load, $V_{mean}$, as the nudging target. The viral load equation becomes


$$\begin{array}{c} \frac{dV}{dt}=N\delta I-cV-\eta (V-V_{mean}), \end{array}$$ (6)

where η is the nudging constant. This approach continuously assimilates the observed data into the model trajectory, pulling the simulated viral load toward the empirical mean. After nudging, we applied a traditional parameter estimation procedure to determine $\omega_{m}$, using the Nelder–Mead algorithm to minimize the loss function


$$\begin{array}{c} L=\frac{1}{n}\sum_{i=1}^{n}{\left[\log\left(V_{model}\right)-\log\left(V_{mean}\right)\right]}^{2}, \end{array}$$ (7)

with the remaining parameters fixed at the values listed in Table 2. The viral load obtained using this estimated $\omega_{m}$ is shown in Fig 6. The viral load data from the 46 individuals is plotted in gray using distinct markers, following the presentation in [84]. The red dashed curve shows the trajectory generated by free virion transmission alone using the value of $k_{m}$ from [81]. The green dash-dot curve shows the trajectory produced by cell-to-cell transmission alone using our estimated $\omega_{m}$. The blue solid curve, which most closely matches the timing of peak viremia, represents the full model incorporating both transmission routes.

Replacing 𝒟(t) with its time average $\bar{𝒟}$, we can compute the reproduction number ℛ via the next-generation matrix method.


$$\begin{array}{c} ℛ=\frac{\beta \bar{𝒟}\left[\delta N𝒦(\mu_{c}+\bar{𝒟})+c𝒲(\mu +\bar{𝒟})\right]}{c\mu (\delta +\mu )(\mu +\bar{𝒟})(\mu_{c}+\bar{𝒟})}. \end{array}$$ (8)

The Malthusian growth parameter *r*, given by the dominant eigenvalue of the Jacobian at the disease-free equilibrium, describes the early exponential growth rate. Both ℛ and *r* characterize thresholds: infection dies out if ℛ<1 (*r* < 0) and is sustained if ℛ>1 (*r* > 0). While ℛ admits a closed-form expression, *r* does not, so we use ℛ to derive threshold conditions for PrEP effectiveness.

**Theorem 1.** *If* ℛ<1, *then* $F_{eff}\geq \overset{0}{\underset{eff}{\bar{F}}}$ *where*


$$\begin{array}{c} \overset{0}{\underset{eff}{\bar{F}}}=\frac{\beta \phi \left(\delta N𝒦+c𝒲\right)-c\mu (\delta +\mu )(\mu_{c}+\phi )}{\phi \left[\beta (\delta N𝒦+c𝒲)-c\mu (\delta +\mu )\right]}. \end{array}$$ (9)

**Proof:** From ℛ<1 we have the inequality


$$\begin{array}{c} c\mu (\delta +\mu )(\mu +\bar{𝒟})(\mu_{c}+\bar{𝒟})>\beta \bar{𝒟}\left[\delta N𝒦(\mu_{c}+\bar{𝒟})+c𝒲(\mu +\bar{𝒟})\right]. \end{array}$$ (10)

Since $\mu_{c}>\mu$,


$$\begin{array}{c} \beta \bar{𝒟}\left[\delta N𝒦(\mu_{c}+\bar{𝒟})+c𝒲(\mu +\bar{𝒟})\right]>\beta \bar{𝒟}\left(\delta N𝒦+c𝒲\right)(\mu +\bar{𝒟}) \end{array}$$ (11)

$$\begin{array}{c} c\mu (\delta +\mu )(\mu_{c}+\bar{𝒟})>\beta \bar{𝒟}\left(\delta N𝒦+c𝒲\right), \end{array}$$ (12)

$$\begin{array}{c} c\mu \mu_{c}(\delta +\mu )>\bar{𝒟}\left[\beta \left(\delta N𝒦+c𝒲\right)-c\mu (\delta +\mu )\right], \end{array}$$ (13)

We replace the term $\bar{𝒟}$ with its constituent definition $\bar{𝒟}\equiv \left(1-{\bar{F}}_{eff}\right)\phi$, then


$$\begin{array}{c} c\mu \mu_{c}(\delta +\mu )>\left(1-{\bar{F}}_{eff}\right)\phi \left[\beta \left(\delta N𝒦+c𝒲\right)-c\mu (\delta +\mu )\right], \end{array}$$ (14)

$$\begin{array}{c} \phi \beta \left(\delta N𝒦+c𝒲\right)-c\mu (\delta +\mu )(\mu_{c}+\phi )<{\bar{F}}_{eff}\phi \left[\beta \left(\delta N𝒦+c𝒲\right)-c\mu (\delta +\mu )\right]. \end{array}$$ (15)

Next we show that βϕ(δN𝒦+c𝒲)−cμ(δ+μ)ϕ>0. Without PrEP, that is, when $\bar{𝒟}=\phi$, ℛ>1. In this situation,


$$\begin{array}{c} \frac{\beta \phi \left(\delta N𝒦(\mu_{c}+\phi )+c𝒲(\mu +\phi )\right)}{c\mu (\delta +\mu )(\mu +\phi )(\mu_{c}+\phi )}>1. \end{array}$$ (16)

Since $\mu_{c}>\mu$,


$$\begin{array}{c} \frac{\beta \phi \left(\delta N𝒦(\mu_{c}+\phi )+c𝒲(\mu_{c}+\phi )\right)}{c\mu (\delta +\mu )(\mu +\phi )(\mu_{c}+\phi )}>\frac{\beta \phi \left(\delta N𝒦(\mu_{c}+\phi )+c𝒲(\mu +\phi )\right)}{c\mu (\delta +\mu )(\mu +\phi )(\mu_{c}+\phi )}, \end{array}$$ (17)

and βϕ(δN𝒦+c𝒲)>cμ(δ+μ)(μ+ϕ)>cμ(δ+μ)ϕ. Thus, the definition for $\overset{0}{\underset{eff}{\bar{F}}}$ in Eq. 9 follows directly from Eq. 15. ◻

Using parameter values from Table 2, $\overset{0}{\underset{eff}{\bar{F}}}=95.5\%$. Referring to Fig 3, $F_{eff}$ declines to ≈97.5% by the end of each injection cycle, after which a new CAB-LA dose raises efficacy to approximately 99.5%. This indicates that drug levels avoid dipping below the threshold for blocking HIV transmission before the next injection, even against drug-sensitive strains. However, the situation becomes more concerning once viral resistance is considered, motivating the analysis in the next section.

**Fig 7:**
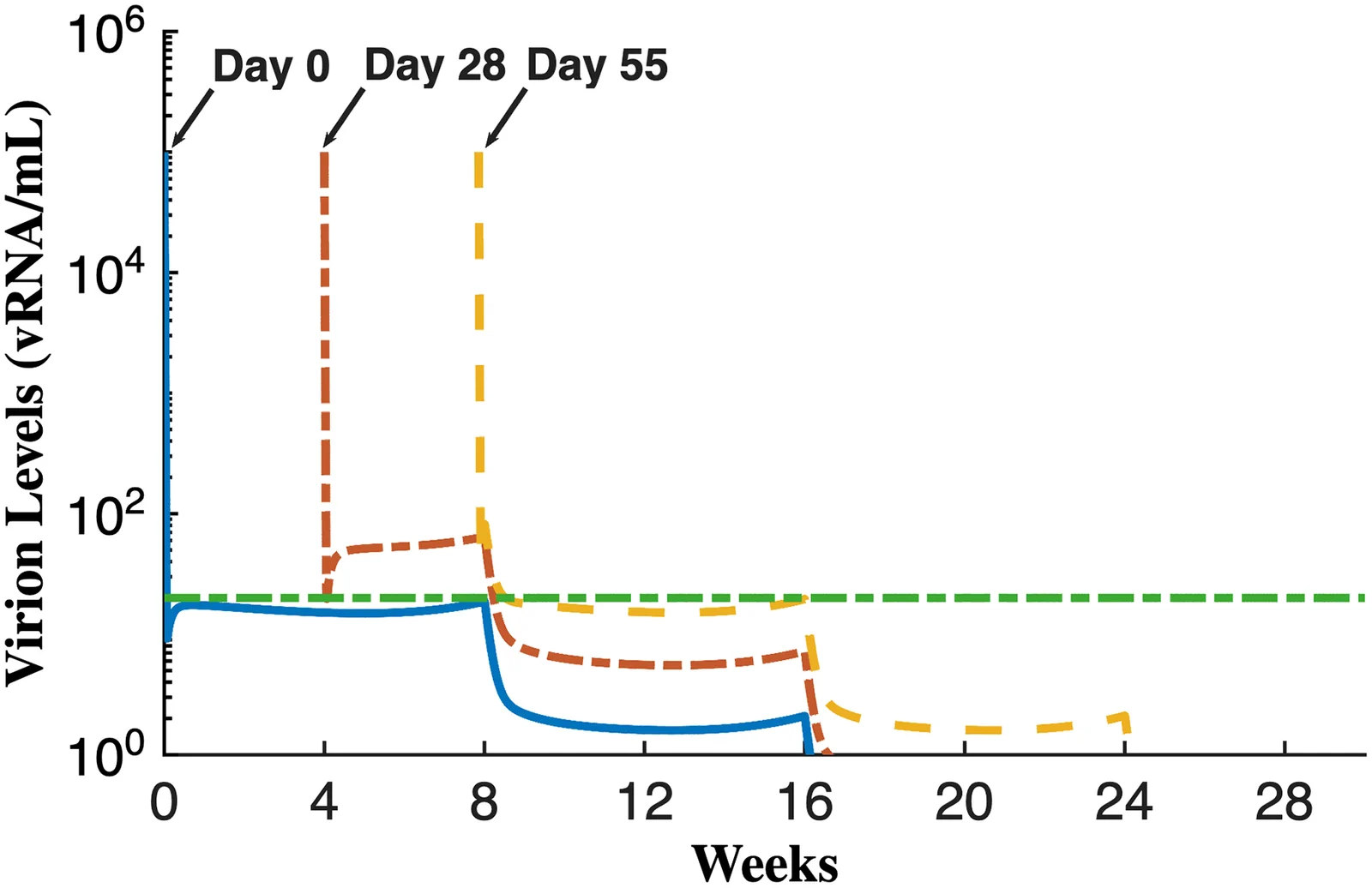
Fully drug-sensitive cellular model of HIV prevention with PrEP. Simulation of T-cell and viral load dynamics over 28 weeks. The third injection occurs at day 0. The blue curve has exposure on day 0, the dot dashed red curve has exposure on day 28, and the gold dashed curve has exposure on day 55. The green line corresponds to the seroconversion threshold used in the simulations to define HIV detectability. Injections are repeated every 56 days. CAB-LA remains effective at preventing establishment of fully drug-sensitive infection for all three exposure days shown.

In Fig 7, we simulate the ODE system over 28 weeks, beginning with the third CAB-LA injection (day 0). Subsequent injections occur every 56 days (8 weeks). HIV exposure of 100 cells/μL occurs on day 0 for the blue curve, on day 28 for the red dot-dashed curve, and on day 55 for the gold dashed curve. These results demonstrate that CAB-LA remains highly effective at preventing the establishment of infection regardless of the day on which the initial exposure occurs. Note that a sharp decline in the viral load occurs at the time of each new CAB-LA injection. The green dashed line in the viral-load plot indicates the seroconversion threshold, at which HIV becomes detectable. In the United States today, seroconversion is most commonly taken to mean an HIV RNA viral load in plasma greater than 20 copies/*mL*, measured by the nucleic acid amplification technology PCR [38,85].

## 8 EGT applied to the cellular model

We apply evolutionary game theory [12] to investigate the effectiveness of CAB-LA in the presence of HIV drug resistance. Following [11], we assume that resistance emerges through Darwinian processes acting on continuous, Gaussian-distributed phenotypic trait *u*. The probability distribution for the trait *u* with variance $\sigma^{2}$ and mean ν is given by the function


$$\begin{array}{c} P(u-\nu )=\frac{1}{\sigma \sqrt{2\pi }}\exp\left(\frac{-(u-\nu {)}^{2}}{2\sigma^{2}}\right). \end{array}$$ (18)

This distribution indicates the relative frequency of infections with the trait *u*. The fraction of these infections successfully suppressed by CAB-LA depends on the drug resistance conferred by the trait *u* and on the time-dependent drug concentration. The mean trait is assumed to be fully drug sensitive and is therefore our reference point ν=0.

The symmetric Gaussian mutation distribution, P(u−ν), centered at ν=0 (fully drug-sensitive), is a mathematical convenience that facilitates analytical tractability within the Cushing–Lande EGT framework [11,12]. This assumption does not conflict with the biological reality that the distribution of mutational *fitness effects* during early HIV infection is typically left-skewed, often approximated by a log-normal distribution, in which the vast majority of mutations are neutral or deleterious and only a small fraction (approximately 5%) confer a measurable fitness advantage [1,2]. We will show how the symmetric mutation distribution can be partitioned into regions corresponding to advantageous and disadvantageous fitness effects and demonstrate that the boundaries of these regions depend on the interaction between drug effectiveness, transmission type, and temporal effects of drug dispersal and declining drug concentrations.

We treat free virion and cell-to-cell infection as distinct evolutionary strategies. The trait $u_{k}$ represents the viral strategy for infection via free virions, while $u_{w}$ represents the strategy for infection via cell-to-cell transfer. The terms $\sigma_{k}^{2}$ and $\sigma_{w}^{2}$ represent the variance of PrEP effectiveness relative to the variance of the infection transmission for each pathway. We rescale $u_{k}$ and $u_{w}$ relative to their respective transmission rate variances so that both have nondimensional magnitudes and no explicit dependence on the variance. Thus, infectivity depends on the traits $u_{k}$ and $u_{w}$ described by the transmission rate per infection type, $𝒦(u_{k})$ and $𝒲(u_{w})$, with the evolutionary parameter definitions given in Table 3. The variances $\sigma_{k}^{2}$ and $\sigma_{w}^{2}$, which appear in ${𝒟}_{f}$ and ${𝒟}_{c}$ respectively, capture how broadly resistance phenotypes are distributed in the viral population. Large $\sigma^{2}$ values imply that CAB-LA remains effective over a wider range of resistant traits, facilitating viral control, while small $\sigma^{2}$ values indicate that even small phenotypic deviations can confer resistance and escape suppression.

**Table 3:** Definition of Evolutionary Parameters.

Transfer	Variable	Definition
Free Virion	$u_{k}$	Free virion transfer trait
	$\sigma_{k}^{2}$	Variance of drug effectiveness relative to the
		variance of the free virion transmission rate
	$s_{k}$	Speed of evolution of free virion transfer trait
Cell-to-cell	$u_{w}$	Cell-to-cell transfer trait
	$\sigma_{w}^{2}$	Variance of drug effectiveness relative to the
		variance of the cell-to-cell transmission rate
	$s_{w}$	Speed of evolution of cell-to-cell virion transfer trait

To distinguish drug effects across pathways, we denote the action of CAB-LA against free virion and cell-to-cell infection by ${𝒟}_{f}$ and ${𝒟}_{c}$, respectively. Small values of ${𝒟}_{f}$ or ${𝒟}_{c}$ indicate strong drug suppression, whereas larger values indicate diminished drug effect as resistance evolves. Both drug terms depend on time, trait value, and variance. The complete trait-dependent transmission and drug-modified progression rates are summarized in Table 4.

**Table 4:** Trait-dependent transmission and drug-modified progression rates.

Transfer	Function	Definition
Free virion	$𝒦(u_{k})=k_{m}\exp\left[-\frac{u_{k}^{2}}{2}\right]$	Transmission rate
	${𝒟}_{f}(t,u_{k},\sigma_{k})=\phi (1-F_{eff}(t)\exp[-\frac{u_{k}^{2}}{2\sigma_{k}^{2}}])$	Drug-modified progression rate
Cell-to-cell	$𝒲(u_{w})=\omega_{m}\exp\left[-\frac{u_{w}^{2}}{2}\right]$	Transmission rate
	${𝒟}_{c}(t,u_{w},\sigma_{w})=\phi (1-F_{eff}(t)\exp[-\frac{u_{w}^{2}}{2\sigma_{w}^{2}}])$	Drug-modified progression rate

The variance parameters $\sigma_{w}^{2}$ and $\sigma_{k}^{2}$ have a specific biological interpretation: they quantify the breadth of drug effectiveness relative to the spread of resistance phenotypes in the viral population. A large $\sigma^{2}$ means that CAB-LA retains substantial inhibitory activity even when the mean viral trait deviates considerably from the drug-sensitive reference (*u* = 0) which corresponds biologically to a drug whose mechanism of action is robust to a wide range of integrase mutations. A small $\sigma^{2}$ means that even modest phenotypic shifts confer substantial resistance, as occurs when resistance mutations produce large structural changes at the drug-binding site. In principle, $\sigma_{k}^{2}$ and $\sigma_{w}^{2}$ could be estimated from in vitro dose-response data for a panel of integrase mutants with known phenotypic resistance levels. Specifically, fitting the drug-modified progression rates $D_{f}$ and $D_{c}$ (Table 4) to the *IC*50 fold-changes observed across a set of resistance mutations would yield empirical estimates of these variances. For integrase inhibitors, fold-change data for mutations at Q148, Y143, and associated pathway mutations are available in the literature (Quashie et al. [41]), and could, in principle, be used to calibrate $\sigma^{2}$ values. We present our results across a range of σ values (0.5, 1, 2) to capture the uncertainty in this quantity, pending experimental characterization.

**Fig 8:**
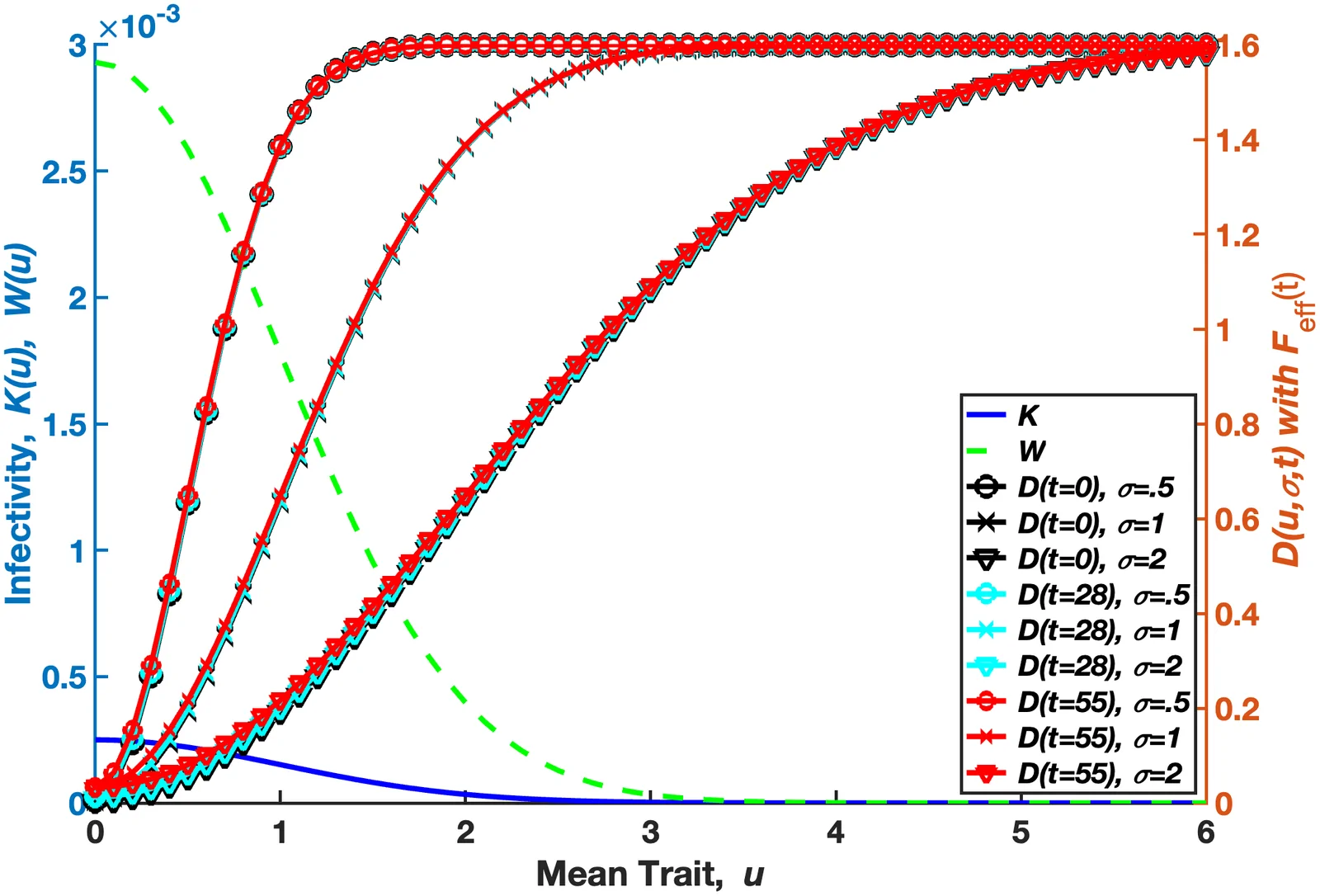
Trait-dependent infectivity and drug suppression. Left: Transmission rates $K(u_{k})$ and $W(u_{w})$ decline as mean traits deviate from zero, reflecting reduced infectivity. Right: Drug-modified progression rates $D_{f}$ and $D_{c}$ vary with trait values and variance parameters $\sigma^{2}$. Results are shown for the post-injection time points *t* = 0, 28, and 55 days and for variance values σ=1 and σ=2, as described in the text. Larger variance corresponds to CAB-LA effectiveness over a wider range of viral traits.

Fig 8 illustrates the relationship between 𝒦, 𝒲, and 𝒟 under constant PrEP levels, with trait values $u_{k}$ and $u_{w}$ varying from 0 to 6. The initial value 𝒲(0) is roughly eleven times greater than 𝒦(0), but both decline rapidly as the mean trait increases. The infectivity 𝒦 (solid blue) approaches zero near u≈2, while 𝒲 (green dashed) becomes negligible around u≈3, indicating that mutations far from the drug-sensitive mean (*u* = 0) are disadvantageous for infection transmission.

The drug progression rate 𝒟 ranges from 0, where the drug fully blocks infection, to 1.6, where it is ineffective. Fig 8 shows 𝒟 at three time points following CAB-LA dosing (*t* = 0, 28, and 55 days) and for two variance values (σ=1 and 2). Differences in σ have a stronger impact on 𝒟 than time. As the variance increases, CAB-LA remains effective across a wider range of viral *t*raits, reflecting greater robustness against resistance.

Because direct experimental data on CAB-LA efficacy against cell-to-cell transmission are not yet available, our model explores a range of scenarios parameterized by the variance $\sigma_{w}$, which captures the degree to which CAB-LA suppresses cell-to-cell transmission as a function of the viral resistance trait. When $\sigma_{w}$ is large, drug action is effective across a wide range of resistant traits; when $\sigma_{w}$ is small, even small phenotypic deviations allow the virus to escape suppression via the cell-to-cell route. Our fitness landscape results (shown in Sections 8.1–8.3) are therefore presented as scenario analyses rather than predictions, bracketing the range of possible biological outcomes consistent with current mechanistic uncertainty. We note that Agosto et al. [71,72] demonstrated that most antiretroviral classes remain effective against cell-to-cell transmission, providing indirect support for at least partial CAB-LA efficacy via this route. We regard this as a central empirical question whose resolution will sharpen the predictive power of the model.

The evolution of the viral traits is modeled by


$$\begin{array}{c} \frac{du_{i}}{dt}=s_{i}\frac{\partial r}{\partial u_{i}}\;\text{ for }i\in \{k,w\}, \end{array}$$ (19)

where *r* is the Malthusian viral fitness and $s_{i}$ is the speed of evolution.

### 8.1 EGT and cell-to-cell transfer only

Following the framework of EGT, we model the dynamics of the mean phenotypic trait $u_{w}$ by assuming that the change in time is proportional to the change in Malthusian fitness as a function of $u_{w}$. The fitness is taken to be the population growth rate (Malthusian growth rate), $r_{c}=r(H,E_{c},I,V;u_{w})$, defined as the spectral bound of the Jacobian of the linearized system [12,86].

The Darwinian system for cell-to-cell transfer only is given by


$$\begin{array}{cc} \frac{dH}{dt} & =\beta -\left[𝒲(u_{w})I+\mu \right]H,\\ \frac{dE_{c}}{dt} & =𝒲(u_{w})IH-\left[{𝒟}_{c}(t,u_{w},\sigma_{w})+\mu_{c}\right]E_{c},\\ \frac{dI}{dt} & ={𝒟}_{c}(t,u_{w},\sigma_{w})E_{c}-(\delta +\mu )I,\\ \frac{dV}{dt} & =N\delta I-cV,\\ \frac{du_{w}}{dt} & =s_{w}\frac{\partial r_{c}}{\partial u_{w}}, \end{array}$$ (20)

where $s_{w}$ is the speed of evolution. Viral control is achieved whenever $r_{c}<0$.

**Theorem 2.** ${ℛ}_{c}(t)\leq 1$ *if and only if* $r_{c}\leq 0$.

**Proof:** Let *J* denote the Jacobian of the system (20) restricted to *H*, $E_{c}$, *I*, and *V*.

The next generation method gives the transition parameter ${\bar{ℛ}}_{c}$ when ${𝒟}_{c}$ is constant.


$$\begin{array}{c} {ℛ}_{c}(t)=\frac{{𝒟}_{c}𝒲\beta }{\mu (\delta +\mu )({𝒟}_{c}+\mu_{c})}. \end{array}$$ (21)

The characteristic polynomial of *J* is


$$\begin{array}{cc} P(\lambda )= & \;(c+\lambda )[\lambda^{2}+\mu (\mu_{c}+{𝒟}_{c}(t,u_{w},\sigma_{w}))+\lambda (\mu +\mu_{c}+{𝒟}_{c}(t,u_{w},\sigma_{w}))-\\ & 𝒲(u_{w}){𝒟}_{c}(t,u_{w},\sigma_{w})\beta /\mu ]. \end{array}$$ (22)

Analysis of the roots yields eigenvalues $\lambda_{1}=-c$, $\lambda_{\pm }=\frac{1}{2}[-\delta -\mu -\mu_{c}-{𝒟}_{c}\pm$ $\sqrt{(\delta +\mu -\mu_{c}-{𝒟}_{c}{)}^{2}+4𝒲(u_{w}){𝒟}_{c}\beta /\mu }]$.

Clearly, $\lambda_{1}<0$ and $\lambda_{-}<0$. Defining $\lambda_{+}=r_{c}$, we obtain


$$\begin{array}{c} r_{c}=\frac{1}{2}\left[-𝒞+\sqrt{{𝒞}^{2}-4𝒲({𝒟}_{c}+\mu_{c})\left(1-{ℛ}_{c}(t)\right)}\right], \end{array}$$ (23)

where $𝒞=\delta +\mu +\mu_{c}+{𝒟}_{c}$.

Thus, $r_{c}\leq 0$ if and only if ${ℛ}_{c}(t)\leq 1$. ◻.

If PrEP is maintained at a constant level, the system admits two disease free equilibria.


$$\begin{array}{c} DFE_{1}=\left(\begin{array}{c} H\\ E_{c}\\ I\\ V\\ u_{w} \end{array}\right)=\left(\begin{array}{c} \frac{\beta }{\mu }\\ 0\\ 0\\ 0\\ 0 \end{array}\right)\;\text{and}\;DFE_{2}=\left(\begin{array}{c} H\\ E_{c}\\ I\\ V\\ u_{w} \end{array}\right)=\left(\begin{array}{c} \frac{\beta }{\mu }\\ 0\\ 0\\ 0\\ u_{w}^{e} \end{array}\right), \end{array}$$ (24)

where $u_{w}^{e}$ satisfies the equilibrium condition


$$\begin{array}{c} \frac{du_{w}}{dt}=0=\frac{-s_{w}u_{w}}{(2r+𝒞)\sigma_{w}^{2}}\left[B({𝒟}_{c}-\phi )+\sigma_{w}^{2}𝒲{𝒟}_{c}\beta /\mu \right], \end{array}$$ (25)

with B=𝒲β/μ−(δ+μ+r).

**Fig 9:**
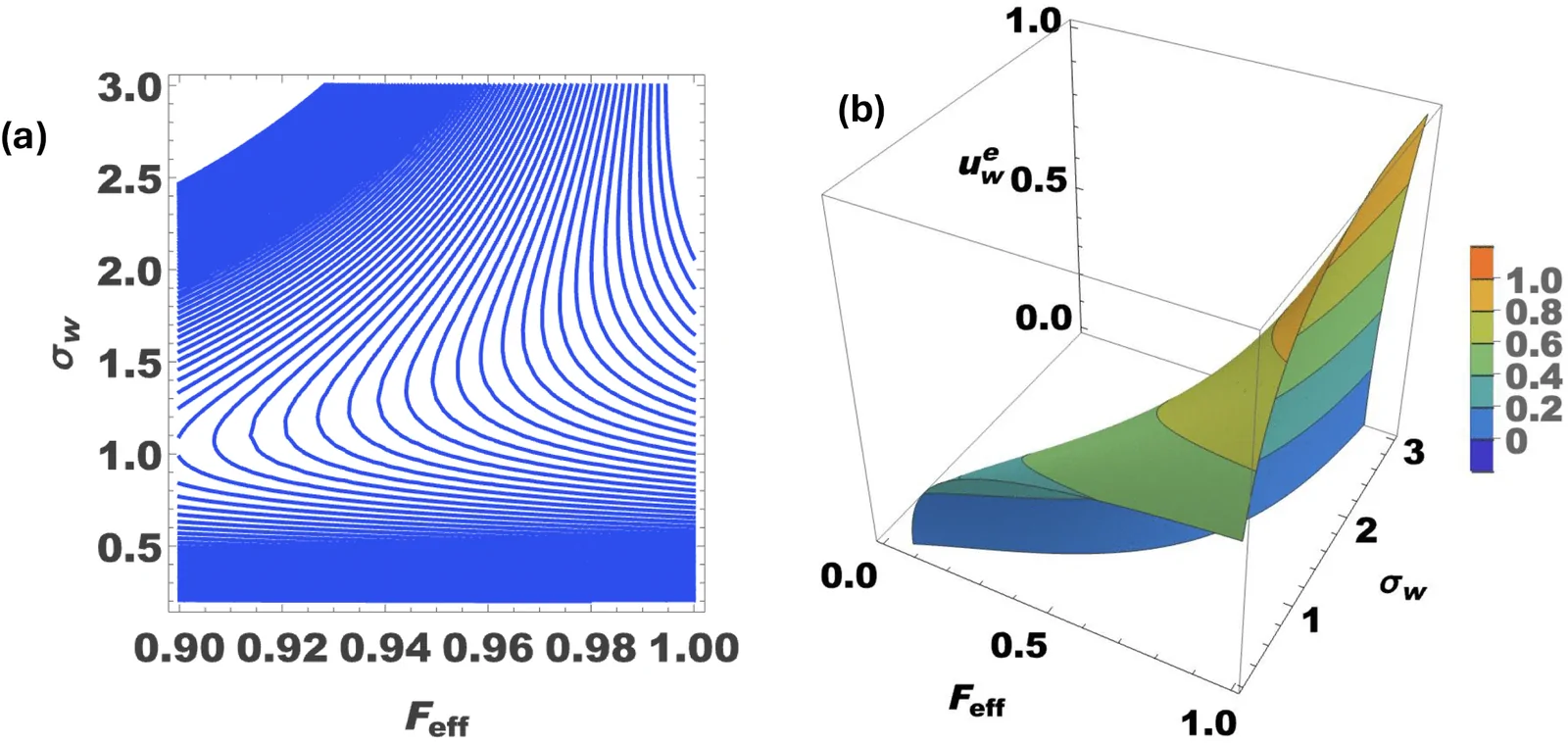
Equilibrium trait values for the cell-to-cell transfer model with trait evolution. (a) Contours of $u_{w}^{e}$ that satisfy the disease free equilibrium conditions as a function of constant drug effectiveness, ${\bar{F}}_{eff}$ and the drug variance, $\sigma_{w}$. (b) The surface represents the equilibrium value $u_{w}^{e}$ as a function of $\sigma_{w}$ and the constant level of PrEP, ${\bar{F}}_{eff}$. The colorbar represents the values of $u_{w}^{e}$.These equilibrium values illustrate the balance between CAB-LA effectiveness and variance needed to maintain the cell-to-cell trait near the fully drug-sensitive value $u_{w}=0$.

This gives the implicit relation


$$\begin{array}{c} B({𝒟}_{c}-\phi )+\sigma_{w}^{2}𝒲{𝒟}_{c}\beta /\mu =0, \end{array}$$ (26)

defining $u_{w}^{e}$ as a function of $\sigma_{w}$ and $\bar{F}eff$. Although no closed-form solution exists, Fig 9 illustrates $u_{w}^{e}$ numerically. In Fig 9a, contour plots show values of $u_{w}^{e}$ consistent with Eq. 26 across drug effectiveness ${\bar{F}}_{eff}$ and variance $\sigma_{w}$. Fig 9b, shows the corresponding surface highlighting that equilibrium traits require a balance between drug effectiveness and variance. Smaller drug effectiveness can be offset by smaller variance, while larger variance necessitates higher drug effectiveness to maintain $u_{w}^{e}$ near the drug-sensitive value $u_{w}=0$. As resistance increases ($u_{w}$ becomes larger), higher drug effectiveness is required to suppress spread. There is a notable fold in the surface appears at low variance, while a wide range of drug effectiveness supports a intermediate values near $u_{w}^{e}\approx 0.6$.

**Fig 10:**
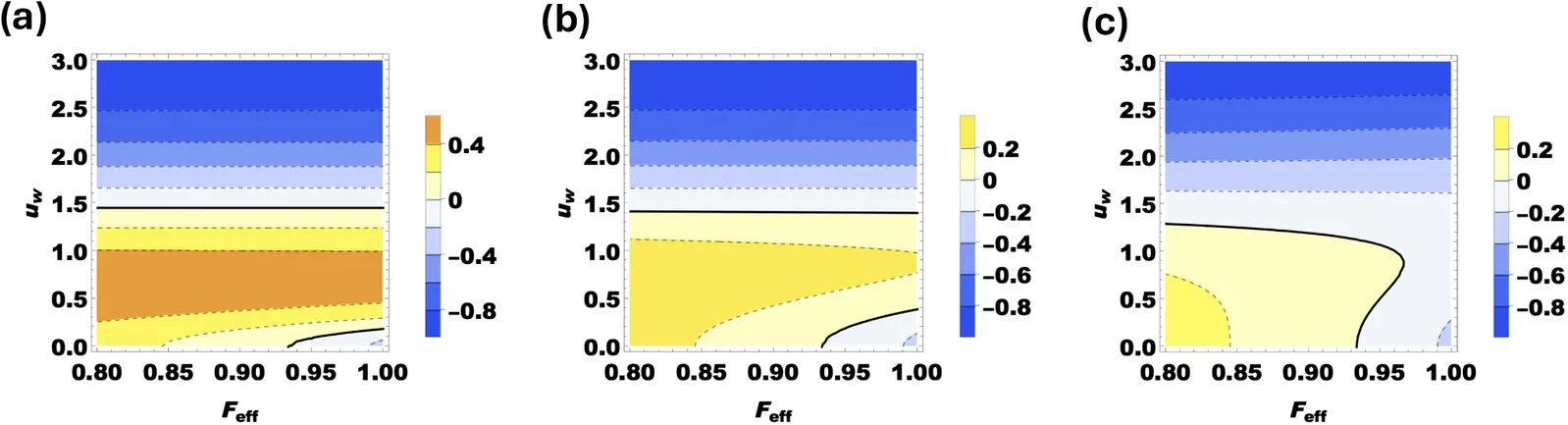
Values of fitness, $r_{c}$, for cell-to-cell transfer model with trait evolution expressed as a function of diminishing $\sigma_{w}$. (a) Heat map of $r_{c}$ values for drug effectiveness, $F_{eff}$, versus mean trait $u_{w}$ for $\sigma_{w}=2$. (b) Heat map of $r_{c}$ values for $F_{eff}$ versus $u_{w}$ for $\sigma_{w}=1$. (c) Heat map of $r_{c}$ values for $F_{eff}$ versus $u_{w}$ for $\sigma_{w}=1/2$. The solid black curves represent $r_{c}=0$. Regions with $r_{c}<0$ indicate viral control, whereas regions with $r_{c}>0$ indicate viral growth. As $\sigma_{w}$ decreases, the negative-fitness region shrinks and higher drug effectiveness is required to maintain control.

The dependence of fitness $r_{c}$ on $u_{w}$, drug effectiveness, and variance is illustrated in heatmaps of the fitness Fig 10 with the time dependence in replaced by the constant drug effectiveness ${\bar{F}}_{eff}$. The black contours ($r_{c}=0$) demarcate the threshold between controlled and growing infections. In each of the subfigures there are two regions where $r_{c}<0$. The first is in the region for $u_{w}$ near the fully drug sensitive mean ($u_{w}=0$) with over 95% drug effectiveness ${\bar{F}}_{eff}$. The second region with $r_{c}<0$ occurs where $u_{w}\approx 1.8$. This drop in fitness occurs due to the drop in infection transmission as the trait evolves far from the drug sensitive mean trait. Each heatmap fixes $\sigma_{w}$ at successively lower values. For $\sigma_{w}=2$ (Fig 10a), fitness decreases with increasing ${\bar{F}}_{eff}$. A region of negative fitness exists for $0\leq u_{w}<0.2$, requiring stronger drug effectiveness as $u_{w}$ increases. For $\sigma_{w}=1$ (Fig 10b), the region of negative fitness shrinks: control is limited to $0\leq u_{w}<0.1$ under high drug effectiveness, while fitness rises steeply at larger $u_{w}$. For $\sigma_{w}=0.5$ (Fig 10c), the negative-fitness region is smaller still, and resistant strains rapidly gain positive fitness as $u_{w}$ grows. Note that as $\sigma_{w}$ shrinks, the fitness reaches higher values in the uncontrolled region, as illustrated by the enlarging orange and red regions.

**Fig 11:**
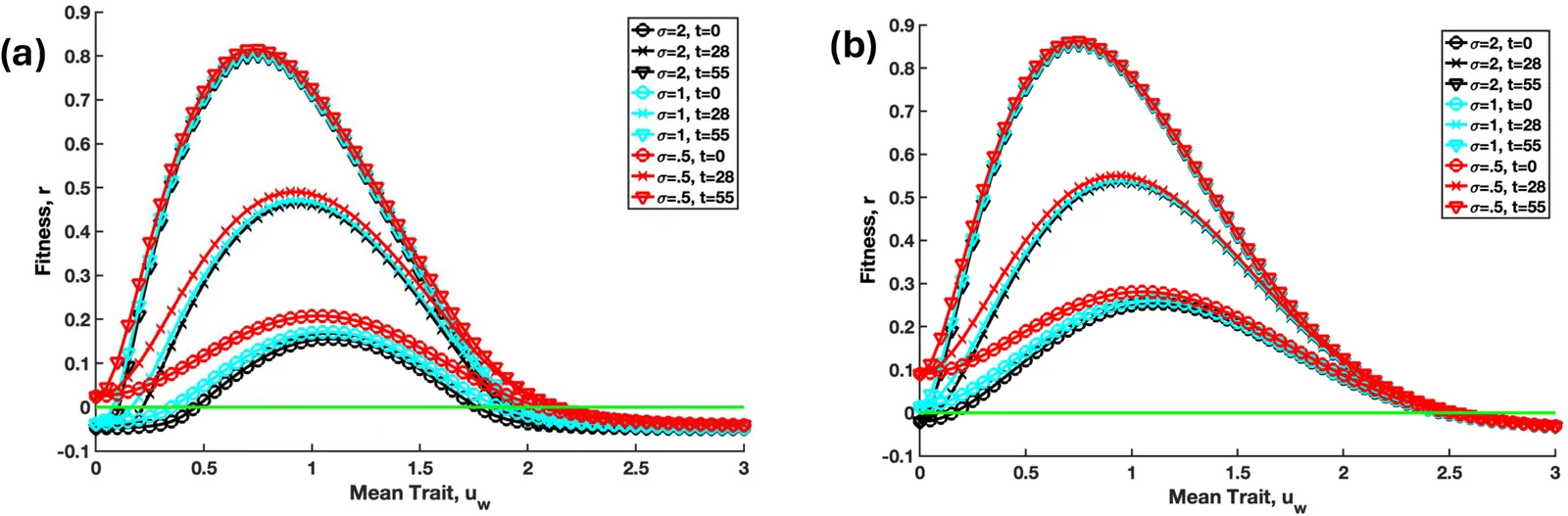
Values of fitness, $r_{c}$, as a function of mean trait, $u_{w}$, for a time-dependent ${ℱ}_{eff}$. Panels compare two fixed values of the free-virion trait: (a) $u_{k}=0$ and (b) $u_{k}=0.5$. The fitness, $r_{c}$, is captured as a function of time (*t* = 0, 28, 55 days after injection) while mean trait $u_{w}$ varies. Results are shown for three representative $\sigma_{w}$ values under time-dependent PrEP efficacy. Curve style denotes time after injection: solid curves indicate *t* = 0, dashed curves indicate *t* = 28 days, and solid curves with circles indicate *t* = 55 days. Negative values of $r_{c}$ indica*t*e that ${ℱ}_{eff}(t)$ is sufficient to prevent HIV spread, whereas positive values of $r_{c}$ indicate viral growth. The black curves represent the fitness function at $\sigma_{w}=2$. The cyan curves are the fitness curves for $\sigma_{w}=1$. The red curves are for $\sigma_{w}=0.5$. The green solid line represents $r_{c}=0$. As CAB-LA effectiveness declines over the injection cycle, $r_{c}$ increases for many $u_{w}$ values; for larger $u_{w}$, fitness can decline because cell-to-cell infectivity $W(u_{w})$ decreases.

Because the concentration of CAB-LA varies over the injection cycle, fitness is also time dependent. Assuming the recommended regimen (two initial injections two weeks apart, followed by injections every 8 weeks), Fig 11 shows the temporal dynamics for $\sigma_{w}=2$ black), $\sigma_{w}=1$ (cyan), and $\sigma_{w}=0.5$ (red). At day zero (immediately post-injection, solid curves), fitness is lowest across all $u_{w}$. At day 28 (mid-cycle, dashed curves), fitness rises, particularly for resistant strains. By day 55 (pre-injection, solid curves with circles), viral fitness exceeds zero for most $u_{w}≲1.4$, including the drug-sensitive case $u_{w}=0$. Interestingly, for large $u_{w}$, fitness drops below zero. This is due to two effects: (i) infectivity $𝒲(u_{w})$ decreases sharply beyond $u_{w}>1$, falling to half its baseline, and (ii) the drug effect 𝒟 ranges from 0 (fully effective) to 1.6 (ineffective), so resistance cannot fully compensate for the transmission cost. The point at which $r_{c}=0$ marks the evolutionary trade-off between drug resistance and transmissibility. This trade-off value of $u_{w}$ depends strongly on the variance $\sigma_{w}^{2}$.

### 8.2 EGT and free virion transfer only

Next, we model the dynamics of the mean phenotypic trait $u_{k}$ by assuming that its temporal change is proportional to the gradient of Malthusian fitness with respect to $u_{k}$. The fitness, $r_{f}=r(H,E_{f},I,V;u_{k})$, is defined as the spectral bound of the Jacobian for the linearized system Darwinian model for free virion transfer only (Eq. 28):


$$\begin{array}{cc} \frac{dH}{dt} & =\beta -\left[𝒦(u_{k})V+\mu \right]H,\\ \frac{dE_{f}}{dt} & =𝒦(u_{k})VH-\left[{𝒟}_{f}(t,u_{k},\sigma_{k})+\mu \right]E_{f},\\ \frac{dI}{dt} & ={𝒟}_{f}(t,u_{k},\sigma_{k})E_{f}-[\delta +\mu ]I,\\ \frac{dV}{dt} & =N\delta I-cV,\\ \frac{du_{k}}{dt} & =s_{k}\frac{\partial r_{f}}{\partial u_{k}}, \end{array}$$ (27)

where $s_{k}$ is the evolutionary rate constant.

We define *J* as the Jacobian by of the subsystem $\left(H,E_{f},I,V\right)$ in (28). The transition parameter $\bar{{ℛ}_{f}}$ when ${𝒟}_{f}$ is constant) can be derived using the next generation method:


$$\begin{array}{c} {ℛ}_{f}(t)=\frac{\beta \delta N𝒦{𝒟}_{f}}{c\mu (\delta +\mu )(\mu +{𝒟}_{f})}. \end{array}$$ (28)

The characteristic polynomial of *J* can be expressed in terms of the transition parameter ${\bar{ℛ}}_{f}$:


$$\begin{array}{c} P(\lambda )=(c+\lambda )(\delta +\lambda +\mu )({𝒟}_{f}+\lambda +\mu )-\delta N𝒦{𝒟}_{f}\beta /\mu . \end{array}$$

**Fig 12:**
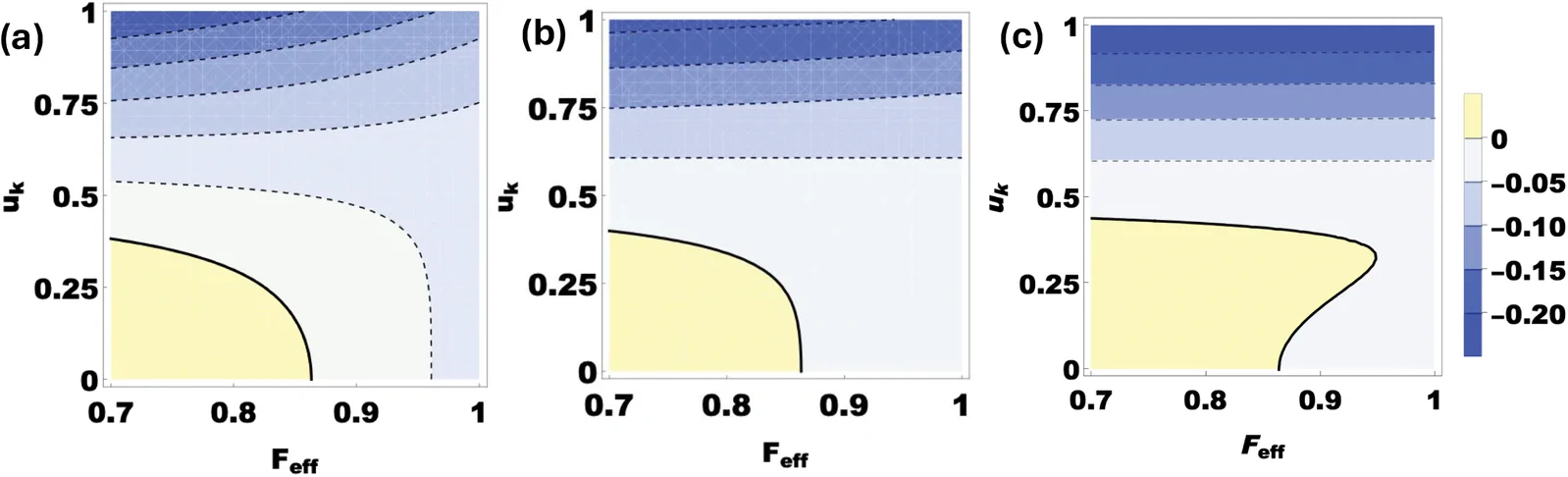
Fitness, $r_{f}$ in the free virion transfer model as a function of mean trait $u_{k}$ and drug effectiveness, for decreasing $\sigma_{k}$. (a) Heatmap of $r_{f}$ for $\sigma_{k}=2$. (b) Heatmap of $r_{f}$ for $\sigma_{k}=1$. (c) Heat map of $r_{f}$ for $\sigma_{k}=1/2$. The solid black curves denote $r_{f}=0$.Regions with $r_{f}<0$ indicate viral control, whereas regions with $r_{f}>0$ indicate viral growth. As $\sigma_{k}$ decreases, the negative-fitness region shrinks and higher drug effectiveness is needed for suppression.

While there is no closed-form solution for the largest eigenvalue $r_{f}$, it can be computed numerically (see Fig 12).

To compute $\partial r_{f}/\partial u_{k}$, we note that since $r_{f}$ is the maximum eigenvalue of *J*, it satisfies $P(r_{f}(u_{k}))=0$. Differentiating with respect to $u_{k}$ yields $\partial P(r_{f}(u_{k}))/\partial u_{k}=0$, which we solve for $\partial r_{f}/\partial u_{k}$ using the value of $r_{f}$ found numerically.

Explicitly,


$$\begin{array}{c} \frac{\partial r_{f}}{\partial u_{k}}=\frac{M_{f}\frac{\partial {𝒟}_{f}}{\partial u_{k}}+M_{k}\frac{\partial 𝒦}{\partial u_{k}}}{L_{f}{𝒟}_{f}+L_{0}}, \end{array}$$ (29)

where


$$\begin{array}{c} \begin{array}{cc} M_{f}=-H_{0}(\delta N𝒦-\tilde{c}(\delta +\tilde{m}), & M_{k}=H_{0}\delta N{𝒟}_{f},\\ L_{f}=(\tilde{c}+\delta +\tilde{m}), & L_{0}=\tilde{m}(\delta +\tilde{m})+\tilde{c}(\delta +2\tilde{m}), \end{array} \end{array}$$ (30)

with $\tilde{c}=c+r$, $\tilde{m}=\mu +r$, and $H_{0}=\beta /\mu$.

The dependence of fitness $r_{f}$ in the free virion transfer model on the mean trait evolution, drug effectiveness, and variance is illustrated in Fig 12. Heatmaps depict $r_{f}$ values. Variance $\sigma_{k}$ is held constant within each panel and decreases from (a) to (c). In Fig 12a with $\sigma_{k}=2$, fitness decreases as drug effectiveness ${\bar{F}}_{eff}$ increases. The solid black curve represents $r_{f}=0$ while the light and medium blue indicate regions where $r_{f}<0$. The yellow regions illustrate $r_{f}>0$. In Fig 12b, with $\sigma_{k}=1$, the region of negative fitness shrinks. When the treatment effectiveness relative to the variance of the infection lowers to 0.5, then higher values of drug effectiveness will be needed to achieve viral control.

### 8.3 EGT with both types of transfer

The full Darwinian system, including both free virus and cell-to-cell transfer is given in system 30.


$$\begin{array}{cc} \frac{dH}{dt} & =\beta -\left[𝒦(u_{k})V+𝒲(u_{w})I+\mu \right]H,\\ \frac{dE_{f}}{dt} & =𝒦(u_{k})VH-\left[{𝒟}_{f}(t,u_{k},\sigma_{k})+\mu \right]E_{f},\\ \frac{dE_{c}}{dt} & =𝒲(u_{w})IH-\left[{𝒟}_{c}(t,u_{w},\sigma_{w})+\mu_{c}\right]E_{c},\\ \frac{dI}{dt} & ={𝒟}_{f}(t,u_{k},\sigma_{k})E_{f}+{𝒟}_{c}(t,u_{w},\sigma_{w})E_{c}-[\delta +\mu ]I,\\ \frac{dV}{dt} & =N\delta I-cV,\\ \frac{du_{k}}{dt} & =s_{k}\frac{\partial r}{\partial u_{k}},\\ \frac{du_{w}}{dt} & =s_{w}\frac{\partial r}{\partial u_{w}}. \end{array}$$ (31)

Here, $s_{k}$ and $s_{w}$ denote the evolutionary speeds of the free-virion and cell-to-cell pathways, respectively.

A closed form explicit analytic expression for *r* is not attainable. However, we can compute $\partial r/\partial u_{i}$ (for *i* = *k*, *w*) by evaluating the eigenvalues of the Jacobian numerically. Specifically, *r* is identified as the maximal real eigenvalue of *J*, satisfying $\det(J-\lambda (u_{k},u_{w})I)=0$, which yields the characteristic polynomial $P(\lambda (u_{k},u_{w}))=0$.

For notational convenience, we define $u=(u_{k},u_{w})$ and $\sigma =(\sigma_{k},\sigma_{w})$. The characteristic polynomial can then be expressed as:


$$\begin{array}{cc} P(\lambda (u))=\; & -\delta N𝒦(u){𝒟}_{f}(u,\sigma )({𝒟}_{c}(u,\sigma )+\lambda (u)+\mu_{c})H_{0}\\ & -(c+\lambda (u))({𝒟}_{f}(u,\sigma )+\lambda (u)+\mu )[{𝒟}_{c}(u,\sigma )𝒲(u)H_{0}\\ & -((\delta +\lambda (u)+\mu )({𝒟}_{c}(u,\sigma )+\lambda (u)+\mu_{c})], \end{array}$$ (32)

where $H_{0}=\beta /\mu$.

Since *r* is the dominant eigenvalue, P(r(u))=0. Differentiating both sides with respect to $u_{i}$ (for *i* = *k* or *w*) yields $\partial P(r(u))/\partial u_{i}=0$, from which $\partial r/\partial u_{i}$ can be solved numerically.

The resulting expression is:


$$\begin{array}{c} \frac{\partial r}{\partial u_{i}}=\frac{C_{f}\frac{\partial {𝒟}_{f}}{\partial u_{i}}+C_{c}\frac{\partial {𝒟}_{c}}{\partial u_{i}}+C_{K}\frac{\partial 𝒦}{\partial u_{i}}+C_{W}\frac{\partial 𝒲}{\partial u_{i}}}{E_{1}{𝒟}_{f}{𝒟}_{c}+E_{f}{𝒟}_{f}+E_{c}{𝒟}_{c}+E_{0}}, \end{array}$$ (33)

with coefficients defined as


$$\begin{array}{cc} C_{f} & =H_{0}(\delta N𝒦({\tilde{m}}_{c}+{𝒟}_{c})+\tilde{c}{𝒟}_{c}𝒲)-\tilde{c}(\delta +\tilde{m})({\tilde{m}}_{c}+{𝒟}_{c})\\ C_{c} & =H_{0}(\delta N𝒦{𝒟}_{f}+\tilde{c}({𝒟}_{f}+\tilde{m})𝒲)-\tilde{c}(\delta +\tilde{m})(\tilde{m}+{𝒟}_{f})\\ C_{K} & =H_{0}\delta N{𝒟}_{f}\left[{\tilde{m}}_{c}+{𝒟}_{c}\right],\\ C_{W} & =H_{0}\tilde{c}{𝒟}_{c}(\tilde{m}+{𝒟}_{f}),\\ E_{1} & =(\tilde{c}+\delta +\tilde{m})-𝒲H_{0}\\ E_{f} & =(\delta +\tilde{m})(\tilde{c}+{\tilde{m}}_{c})+\tilde{c}{\tilde{m}}_{c}-\delta N𝒦H_{0}\\ E_{c} & =(\delta +\tilde{m})(\tilde{c}+\tilde{m})+\tilde{c}\tilde{m}-(\tilde{c}+\tilde{m})H_{0}𝒲\\ E_{0} & =\tilde{m}(\delta +\tilde{m})(\tilde{c}+{\tilde{m}}_{c})+\tilde{c}(\delta +2\tilde{m}){\tilde{m}}_{c} \end{array}$$ (34)

where $\tilde{c}=c+r$, $\tilde{m}=\mu +r$, ${\tilde{m}}_{c}=\mu_{c}+r$, and $H_{0}=\beta /\mu$.

**Fig 13:**
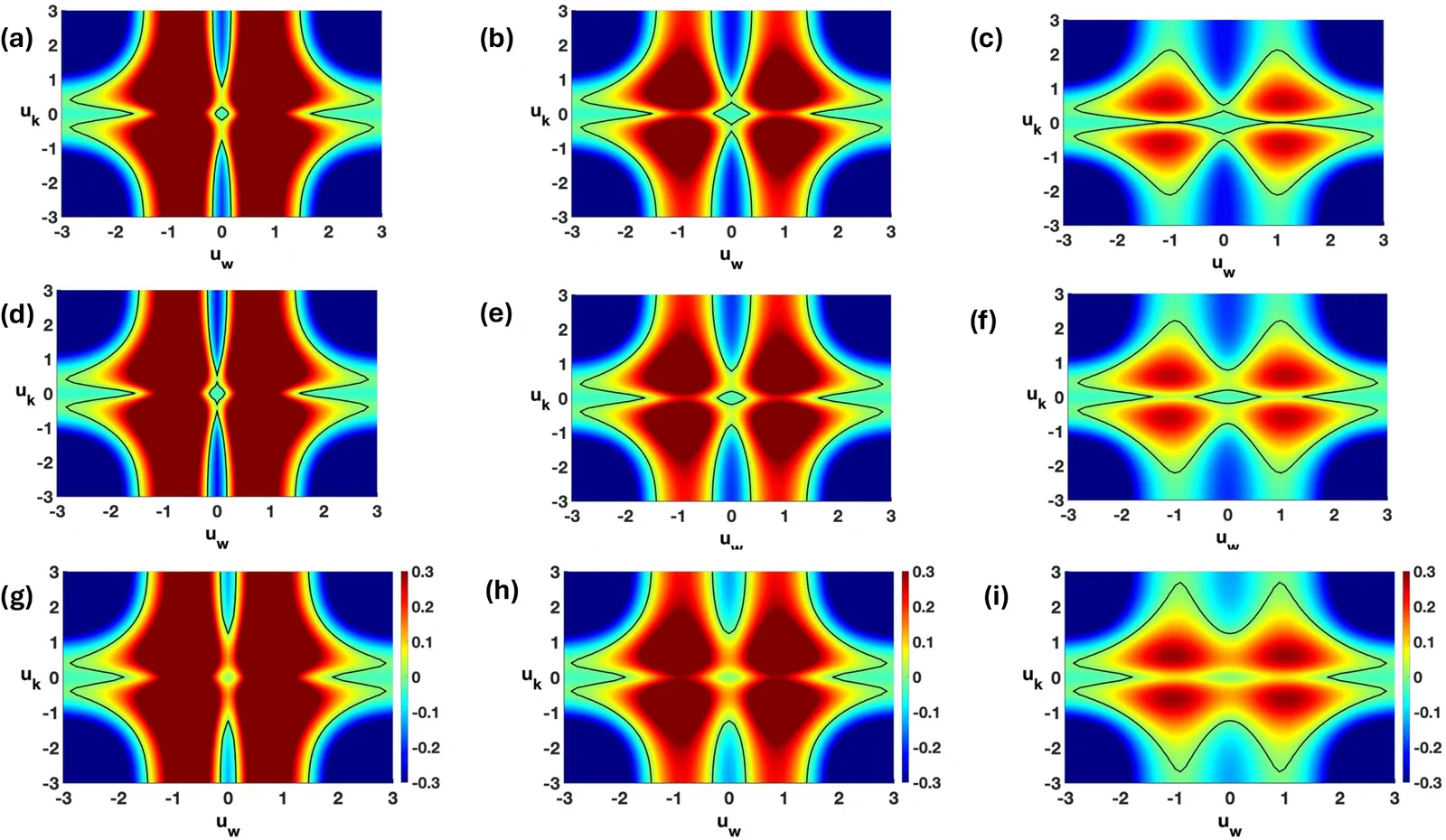
Heatmap of the fitness, *r*, as a function of the evolving traits for $\sigma_{k}=0.5$. Heatmaps show the Malthusian fitness *r* as a function of the free-virion trait $u_{k}$ and the cell-to-cell trait $u_{w}$, with $\sigma_{k}=0.5$ fixed in all panels. The first row represents the fitness on the day of the injection, *t* = 0 (top row; immediately after injection). The middle row represents halfway through the cycle between injections at *t* = 28 days (middle row). The third row is the day before the next injec*t*ion, *t* = 55 (bottom row). The value of the drug variance with respect to free virion transfer is constant in each hea*t*map at $\sigma_{k}=0.5$. The drug variance with respect to the cell-*t*o-cell transfer increases in each column from left to right with $\sigma_{w}=0.5$, to 1, and 2. The black contour marks *r* = 0; regions with *r* < 0 indicate CAB-LA-mediated viral suppression, whereas regions with *r* > 0 indicate viral growth under drug pressure.

**Fig 14:**
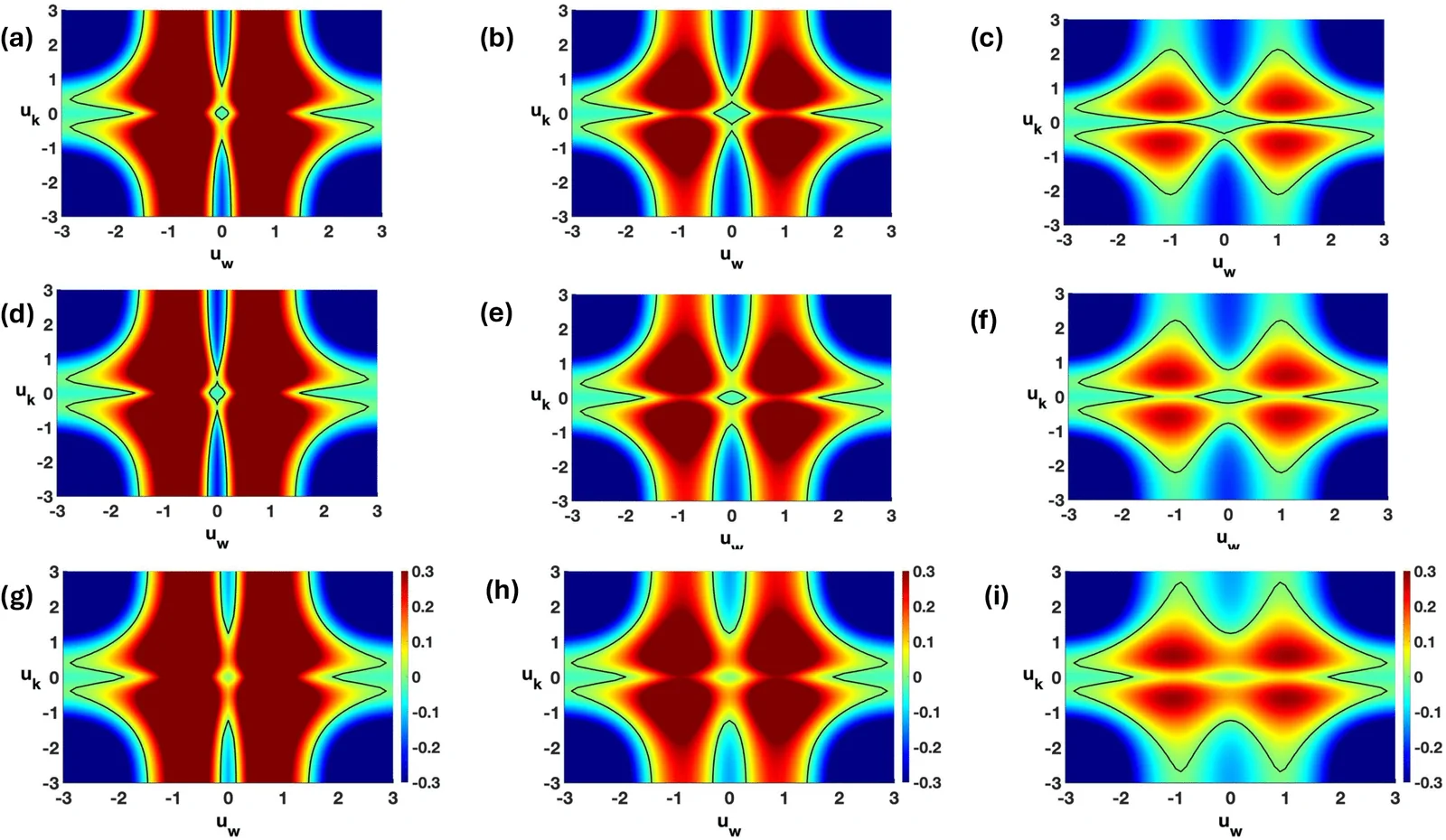
Fitness seascapes with broad free-virion drug-effectiveness coverage. Heatmaps show the Malthusian fitness *r* as a function of the free-virion resistance trait $u_{k}$ and the cell-to-cell resistance trait $u_{w}$, with $\sigma_{k}=2$ fixed in all panels. The first row represents the fitness on the day of the injection, *t* = 0 (top row; immediately after injection). The middle row represents halfway through the cycle between injections at *t* = 28 days (middle row). The *t*hird row is the day before the next injection, *t* = 55 (bottom row). Columns show increasing cell-to-cell drug-effectiveness coverage, with $\sigma_{w}=0.5$, 1, and 2 from lef*t* to right. The black contour marks *r* = 0; regions with *r* < 0 indicate viral con*t*rol by CAB-LA, whereas regions with *r* > 0 indicate viral growth or escape. Increasing $\sigma_{w}$ expands the negative-fitness region, indicating broader control of cell-to-cell resistance traits.

Figs 13 and 14 illustrate the resulting fitness landscapes as functions of the evolving mean traits in both the free-virion and cell-to-cell transmission pathways. Each subfigure provides a snapshot of the fitness, *r*, as a function of the mean traits $u_{k}$ and $u_{w}$ at a specific time point. Taken together, the sequence of snapshots for each variance level reveals the underlying evolutionary tradeoff: mutations must deviate sufficiently from the fully drug-sensitive trait to evade CAB-LA-mediated suppression, but not so far that they substantially impair the ability of the virus to infect healthy T-cells.

Because *r* < 0 indicates that CAB-LA successfully suppresses infection, regions of the fitness landscape with negative fitness correspond to disadvantageous mutational fitness effects. Conversely, regions with *r* > 0 correspond to advantageous mutational fitness effects, where viral strains can persist despite drug pressure. The figures also illustrate how the boundaries between these regions shift over time in response to changing drug concentrations, revealing how the evolutionary opportunities for resistance expand and contract as CAB-LA levels rise and subsequently decline.

Importantly, both figures demonstrate that although the mutation kernel is modeled by a symmetric Gaussian distribution, the resulting distribution of mutational fitness effects is generally asymmetric. The nonlinear interaction between drug effectiveness, transmission pathway, and viral infectivity creates fitness landscapes in which advantageous and disadvantageous mutations occupy unequal regions of trait space. Thus, a symmetric distribution of mutational changes can produce a highly skewed distribution of fitness effects.

In both Figs 13 and 14, the variance of drug effectiveness against cell-to-cell transfer, $\sigma_{w}$, is held constant across each column. Column 1 uses $\sigma_{w}=0.5$, increasing to 1 in column 2, and 2 in column 3. The first row displays fitness on day 0 (immediately after the CAB-LA injection, when plasma concentration is at its peak). The middle row shows day 28, halfway through the dosing interval as drug levels wane. The third row shows day 55, the day before the next injection, when CAB-LA concentration is at its lowest. In each subfigure, the black curve marks the *r* = 0 contour, and blue regions (from light to dark) indicate negative fitness, i.e., drug levels are sufficient to achieve viral suppression.

Fig 13 fixes the variance of drug effectiveness for free-virion transfer at $\sigma_{k}=0.5$ while $\sigma_{w}$ varies from 0.5 to 2. On days 0 and 28, a small region near $\left(u_{k},u_{w})=(0,0\right)$ exhibits negative fitness, and this region enlarges as $\sigma_{w}$ increases. Additional pockets of negative fitness appear when $u_{k}$ reaches magnitude 1 for $u_{w}$ near 0. The regions of positive fitness shrink as $\sigma_{w}$ grows, indicating that broader drug action against cell-to-cell resistance reduces opportunities for viral escape.

Fig 14 holds $\sigma_{k}=2$ while $\sigma_{w}$ again varies from 0.5 to 2. In this regime, a band of negative fitness appears for all values of $u_{k}$ when $u_{w}$ is near 0 for the first half of the dosing cycle. Positive-fitness regions contract dramatically as $\sigma_{w}$ increases, demonstrating that when PrEP acts broadly against mutations, viral growth is substantially suppressed.

Across all subfigures, when $|u_{w}|\approx 2.5$ the fitness becomes negative; in this region, mutations degrade the ability of the virus to infect new cells via cell-to-cell transfer, making them evolutionarily detrimental. In the center of each subfigure, a thin region, corresponding to small deviations from the fully drug-sensitive trait, remains near the fitness-neutral contour *r* = 0. On day 0 (top row), CAB-LA is sufficiently concentrated to prevent viral spread even for these small mutations, as indicated by the blue region bordered by the *r* = 0 curve. By day 28 (middle row), this central region of negative fitness shrinks, revealing the tradeoff in the free-virion pathway: as $u_{k}$ evolves toward values near $|u_{k}|\approx 1$, the virus can partially evade CAB-LA but becomes less able to infect via free virions. When $u_{w}$ remains small, the combination of decreasing free-virion infectivity and ongoing PrEP effectiveness prevents viral escape.

By day 55 (last row), however, plasma CAB-LA concentration is low, and the central region becomes fitness-positive. At this point, cell-to-cell mutations provide enough resistance to drive infection even when the free-virion trait has evolved to a state where infectivity is impaired. The only consistently deleterious mutations across all scenarios are those with $|u_{w}|\approx 2.5$ or greater.

## 9 Evolution of traits over time

**Fig 15:**
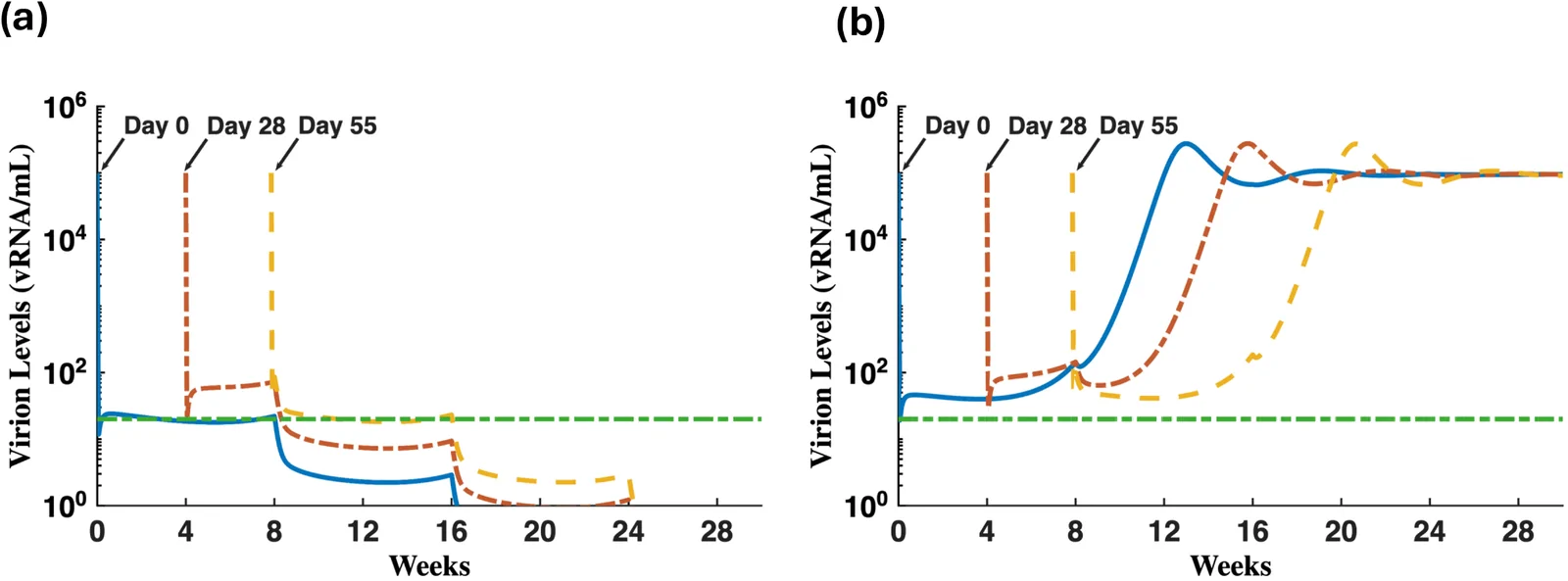
Comparison of viral load across HIV exposure days. HIV Exposure on day 0, day 28, or day 55 following the most recent CAB-LA injection. The mean traits start at $u_{k}=u_{w}=0.1$ with (a) a less effective drug response to mutation, $\sigma_{k}=\sigma_{w}=1$, and (b) a more effective drug response to mutation, $\sigma_{k}=\sigma_{w}=2$. In panel (a), PrEP is insufficient to prevent viral growth for all three exposure days. In panel (b), PrEP controls infection for all three exposure days. Thus, within the cases shown, the day of exposure does not change the final outcome. The horizontal green dashed line marks the seroconversion threshold used in the simulations.

Before we study the evolution of the free virion and cell-to-cell transfer traits, $u_{k}$ and $u_{w}$ respectively, we must investigate whether the timing of HIV exposure in the CAB-LA cycle is important. In Fig 15 we illustrate the effects of exposure on days 0, 28, or 55. Fig 15(a) shows a less effective drug response to mutation with $\sigma_{k}=\sigma_{w}=1$. In this case, PrEP is insufficient to prevent HIV infection from growing in all three exposure scenarios. Fig 15(b) shows a more effective drug response to mutation with $\sigma_{k}=\sigma_{w}=2$. Here, regardless of the day of exposure, PrEP is effective.

In both cases shown in (a) and (b), the day of exposure does not change the end result. Thus in this section we will assume that the HIV exposure occurs on day 0 of the CAB-LA injection cycle.

**Fig 16:**
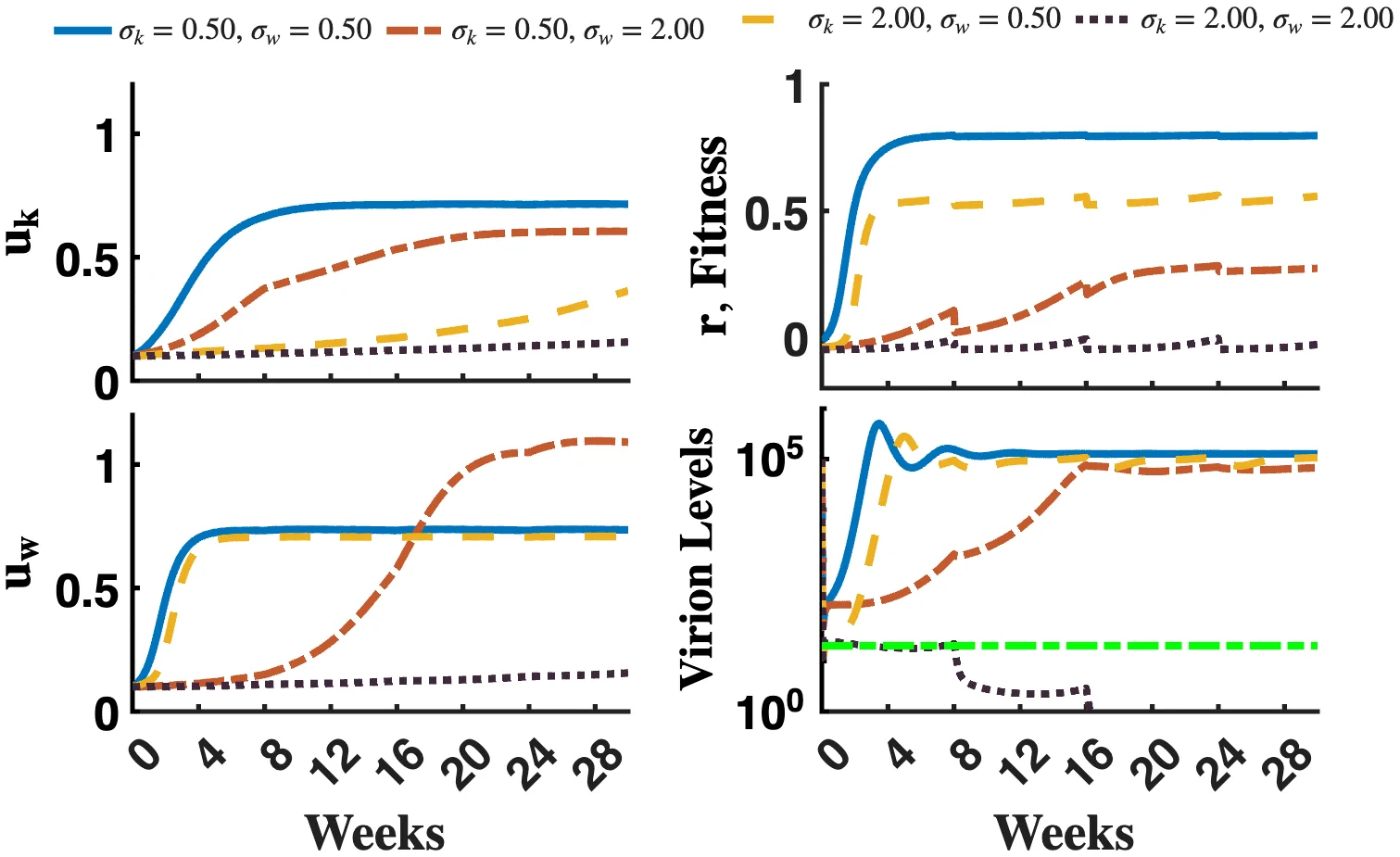
Evolution after HIV exposure with starting traits $u_{k}=u_{w}=0.1$. The blue solid curve represents $\sigma_{k}=\sigma_{w}=0.5$, the orange dash dot curve represents $\sigma_{k}=0.5$ and $\sigma_{w}=2$, the gold dashed curve represents $\sigma_{k}=2$ and $\sigma_{w}=0.5$, and black dotted curve represents $\sigma_{k}=\sigma_{w}=2$. HIV exposure occurs on the day of the third PrEP injection, labeled *t* = 0. The left column shows the evolution of the free-virion trait $u_{k}$ and cell-to-cell trait $u_{w}$; the right column shows fitness and viral load over time. Broad drug-effectiveness coverage against both traits, $\sigma_{k}=\sigma_{w}=2$, leads to viral clearance, whereas the other cases shown escape CAB-LA. The horizontal green dashed line marks the seroconversion threshold used in the simulations.

Next, we investigate how the free virion transfer and cell-to-cell transfer traits evolve in response to varying levels of drug effectiveness against mutations. In Fig 16, HIV is introduced into the system on the day of the third PrEP injection corresponding to the time of maximal drug protection. We label HIV exposure as day *t* = 0.

Fig 16 depicts the temporal evolution of the drug resistant traits $u_{k}$ and $u_{w}$ for an exposure strain possessing initial resistance of $u_{k}=u_{w}=0.1$, which is close to the fully drug sensitive case. The left column shows the evolution over time of the free virion transmission trait $u_{k}$ (top) and cell-to-cell transmission trait $u_{w}$ (bottom). The right column displays fitness (top) and the virion load (bottom) as functions of time. Each PrEP injection appears as a cusp in the fitness and virion trajectories, reflecting abrupt increases in drug concentration.

When the drug effectiveness coverage for both traits is narrow, $\sigma_{k}=\sigma_{w}=0.5$ (blue curve), the traits quickly evolve to stable values around 0.7. Correspondingly, both fitness and virion levels grow quickly and stabilize.

The green dashed line in the viral levels plot indicates the seroconversion threshold. Examining the virion load, we observe that the infection in this simulation is fully controlled when $\sigma_{k}=\sigma_{w}=2$, while all the other cases escape CAB-LA by week 8.

Fig 16 demonstrates that the evolution of each trait depends on the variances in the drug effectiveness against mutation. While both traits evolve away from the fully drug sensitive ($u_{k}=u_{w}=0$), the traits associated with free virion and cell-to-cell transfer follow distinct evolutionary trajectories. Larger variance values correspond to broader and more effective coverage against the mutation. However, in the $u_{w}$ subfigure, the red dash dot and black dotted curves, both corresponding to broad coverage against cell-to-cell transfer mutations with $\sigma_{w}=2$, exhibit different evolutionary behavior due to differences in coverage against free virion transfer mutations. The red dash dot curve corresponds to a narrow coverage against free virion trait mutations ($\sigma_{k}=0.5$). In this case, the virus escapes PrEP. As fitness increases, the viral load also rises. In contrast, the black dotted curve has a broad coverage against mutation for both traits ($\sigma_{k}=\sigma_{w}=2$). Here, the fitness remains close to zero and viral clearance is achieved.

Next, we compare the cases with broad coverage against mutations for the free virion trait, $\sigma_{k}=2$, represented by the gold dashed and black dotted curves. The gold dashed curve has narrow coverage against mutations in the cell-to-cell transfer and results in viral escape. The black curve has broad coverage and achieves viral clearance. Under narrow cell-to-cell coverage (gold dashed curve), the trait $u_{w}$ evolves rapidly while the free-virion trait evolves more slowly. The result is a rapid increase in fitness and delayed seroconversion compared to the case with narrow coverage for both traits (blue curve). Although three of the scenarios shown in Fig 16 result in viral escape, the timing of seroconversion differs across cases. Finally, while the two traits correspond to distinct transmission mechanisms, Fig 16 demonstrates that their evolutionary dynamics are coupled.

The decaying behavior of the virion load in Fig 7 for $\sigma_{k}=\sigma_{w}=2$ (black dashed curve) can be predicted by examining the third column of Fig 14 (subfigures (c), (f) and (i)). As shown in these heatmaps, the traits satisfying $u_{k}\leq 0.5$ and $u_{w}\leq 0.5$ have negative fitness for *t* = 0 and *t* = 28. By day 55, the fitness is positive, but remains small, so another CAB-LA injection is sufficient to maintain control. In con*t*ras*t*, as seen in the first and third columns of Fig 13 and first column of Fig 14, when one or both of the variances is 1 or smaller, the region of negative fitness shrinks to $u_{k}\leq 0.1$, $u_{w}\leq 0.1$. By day 55, the fitness is large and positive for values near $u_{k}=u_{w}=0.1$. Thus, we can predict that CAB-LA will fail to control the HIV exposure for the case of $\sigma_{k}=\sigma_{w}=2$, $\sigma_{k}=0.5$ and $\sigma_{w}=2$, or $\sigma_{k}=2$ and $\sigma_{w}=0.5$.

**Fig 17:**
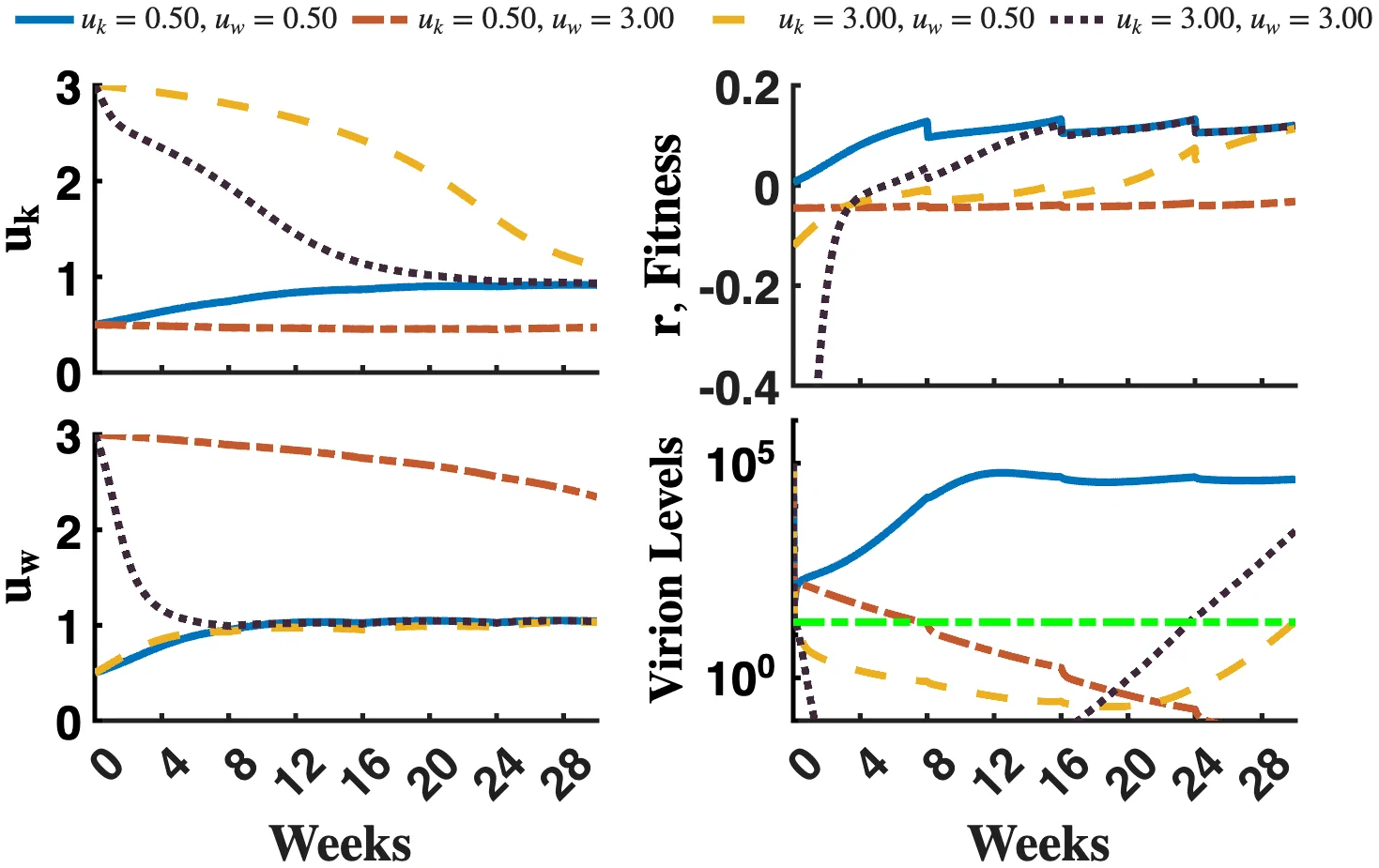
Evolution after HIV exposure with variance $\sigma_{k}=\sigma_{w}=2$. The blue solid curve represents $u_{k}=u_{w}=0.5$, the orange dash dot curve represents $u_{k}=0.5$ and $u_{w}=3$, the gold dashed curve represents $u_{k}=3$ and $u_{w}=0.5$, and black dotted curve is $u_{k}=u_{w}=3$. These simulations compare how different initial trait configurations affect evolutionary escape when drug-effectiveness coverage is broad for both transmission pathways. Evolutionary escape occurs for some initial trait configurations despite $\sigma_{k}=\sigma_{w}=2$, while the case $u_{k}=0.5$ and $u_{w}=3$ remains in a region of diminished infectivity and is controlled by CAB-LA. The horizontal green dashed line marks the seroconversion threshold used in the simulations.

Next, we investigate cases with fixed variances $\sigma_{k}=\sigma_{w}=2$ and starting trait values of 0.5 or 3. For each case, Fig 14 predicts CAB-LA will control the infection. When $u_{k}=0.5$ or 3, the infectivity via free virion transfer is initially too diminished to allow virion growth. However, as shown in Fig 17, evolutionary escape occurs for the initial combination $u_{k}=u_{w}=0.5$ (blue curve). After 28 weeks, the starting trait combinations $u_{k}=3$, $u_{w}=0.5$ (gold dashed curve) and $u_{k}=3$, $u_{w}=0.3$ (black dotted curve) evolve sufficiently to allow the free virion level to reach seroconversion levels. Notably, the timing of seroconversion differs substantially among these cases. In contrast, the starting case $u_{k}=0.5$ and $u_{w}=3$ (red dash dot curve) trait evolution does not escape the region of diminished infectivity. Hence, CAB-LA is sufficient to block viral growth for this initial trait value combination.

## 10 Delay in HIV diagnosis

In the CAB-LA trial, HPTN 083, HIV detection was delayed in 11 of 16 infections among participants receiving CAB-LA injections [37]. Four of the participants in the HPTN 083 were positive at the trial enrollment. Detection of these HIV infection was delayed with a mean delay 62 of days. For those who were infected during the CAB-LA trial, 7 of the 12 HIV incident cases had a mean delay of 98 days.

Liegeon et al. [38] noted that the majority of these infections would have been detected at an earlier visit using a nucleic acid test (NAT) antigen test. While our numerical study does not capture antigen development, we do capture seroconversion at 20 copies/mL.

**Fig 18:**
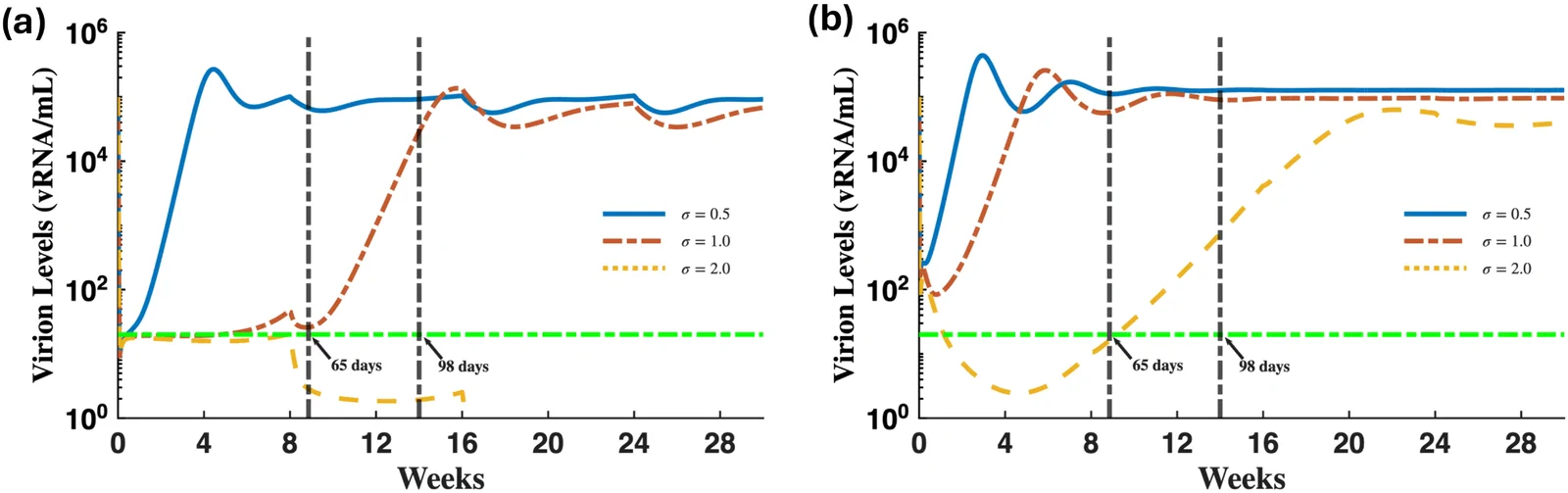
Two scenarios for delayed seroconversion. The delay window of 62 to 98 days is designated by the two vertical black lines. Seroconversion is estimated at 20 copies/mL. Panel (a) shows delayed seroconversion for starting trait values $u_{k}=2$ and $u_{w}=0.2$ under broad drug-effectiveness variance, with comparison to a more moderate variance case. Panel (b) shows starting trait values $u_{k}=0$ and $u_{w}=0.12$, where broad drug-effectiveness variance controls HIV but moderate variance, $\sigma_{k}=\sigma_{w}=1$, produces seroconversion between days 62 and 98. The horizontal green dashed line marks the seroconversion threshold used in the simulations. A lower detection threshold would shift the predicted threshold-crossing time earlier.

In Fig 18 the 62nd and 98th days are designated by black vertical lines. Delayed seroconversion reached between days 62 and 98 in multiple combinations of traits and variances in drug effectiveness. The first example case is shown in Fig 18(a) where the starting trait values reflecting mixed infectivity, $u_{k}=2$ and $u_{w}=0.2$ along with a broad drug effectiveness reflected by $\sigma_{k}=\sigma_{w}=2$. HIV is uncontrolled in this case with a significantly delayed seroconversion while a more moderate variance in drug effectiveness results in a delay of only 2–3 weeks. The second example case is illustrated in (b) with low starting value trait values reflecting infectivity close to that of the fully drug-sensitive strain, $u_{k}=0$, $u_{w}=0.12$. While a broad drug effectiveness controls HIV, a moderate variance in drug effectiveness with $\sigma_{k}=\sigma_{w}=1$, fails with seroconversion occurring between days 62 and 98.

To assess robustness of our conclusions to parameter uncertainty, we performed additional simulations varying the free-virion infection rate $k_{m}$ (±50%), the cell-to-cell infection rate $\omega_{m}$ (±50 %), the drug *IC*50 (via $F_{eff}$, ±10 %), and the viral burst size *N* (±50 %). In all cases, the threshold structure, specifically the existence of a minimum drug effectiveness $\overset{0}{\underset{eff}{\bar{F}}}$ below which infection is not controlled, and the qualitative ordering of evolutionary outcomes across σ values was preserved. The timing of seroconversion in Fig 18 shifted by 5–15 days under the largest parameter perturbations, but the 62–98 day delay window consistent with clinical trial observations remained achievable across all tested parameter combinations. These results suggest that the main conclusions are robust to the parameter uncertainty inherent in a first-principles model of this kind.

These results indicate that delayed seroconversion is a result of the trade-off between drug-resistance and infectivity. This trade-off generates nontrivial evolutionary trajectories, including the temporary viral suppression followed by rebound as shown in Fig 18.

## 11 Conclusion

We have developed an evolutionary game theory framework to study the emergence of drug resistance following HIV exposure while on CAB-LA. By explicitly coupling within-host HIV dynamics with PKPD and the dual transmission pathways, we constructed a time-dependent fitness seascape that captures the evolutionary consequences of fluctuating drug concentrations. This approach extends classical within-host HIV models by allowing drug resistance traits to evolve continuously in response to dynamic selective pressure imposed by bimonthly cabotegravir injections.

Our results demonstrate that CAB-LA can prevent the establishment of infection when the drug effectiveness remains sufficiently broad against resistance traits. In the absence of drug resistance, CAB-LA maintains viral suppression throughout the dosing interval, consistent with clinical trial observations. However, when the drug-resistance traits are allowed to evolve, the outcomes are sensitive to the variance of drug effectiveness relative to mutational effects on infectivity. Narrow drug effectiveness profiles permit rapid evolutionary escape, while broader profiles can suppress viral growth even in the presence of evolving resistance and consequent variable infectivity. These findings highlight the critical role of evolutionary trade-offs between drug resistance and the viral ability to infect healthy T-cells. In addition, the results indicate a greater capacity to evade the pharmacological inhibition of CAB-LA as compared to free virion transfer.

The fitness landscape analysis reveals that the fluctuating drug concentrations reshape selective pressures over each injection cycle, producing transient windows in which resistant strains may gain a fitness advantage. Nevertheless, the mutations that strongly reduce infectivity ultimately become deleterious, creating an upper bound on viable drug-resistance. This trade-off generates nontrivial evolutionary trajectories, including temporary viral suppression followed by rebound, and helps explain observed delays in HIV detectability under CAB-LA. Our model provides a mechanistic explanation for delayed seroconversion reported in clinical trials, linking pharmacological suppression, early viral dynamics, and resistance evolution.

Quantitative validation against individual-level patient data, such as serial viral load and genotypic resistance testing from HPTN 083 or 084 [13,14] participants who acquired infection, was not possible because these data are not publicly available. Such data, particularly time-resolved viral load trajectories and resistance genotypes from breakthrough infections, would allow the resistance trait evolution predicted in Figs 16 and 17 to be directly tested and would enable formal parameter fitting for the evolutionary speed constants $s_{k}$ and $s_{w}$, which are currently treated as free scaling parameters.

Our model focuses on the acute phase of HIV infection and does not include a latent reservoir compartment. In the acute setting, viral dynamics are dominated by actively replicating cells, and the exclusion of latency is consistent with our time horizon of 28 weeks. However, the latent reservoir becomes clinically important for longer-term resistance predictions. T-cells infected via cell-to-cell transfer have a greater propensity for latency [74], and latently infected cells can harbor archived resistance mutations that re-emerge during treatment interruption or during the extended pharmacological tail of CAB-LA. Incorporating a latent compartment in future extensions of this framework would be valuable for assessing the long-term persistence of resistance mutations and their impact on subsequent antiretroviral therapy.

Although we focus on CAB-LA throughout, the Darwinian fitness seascape framework developed here is broadly applicable to antiretroviral agents with time-varying pharmacokinetics. The framework requires three structural components: (1) a PK/PD model that produces a time-dependent drug effectiveness function $F_{eff}(t)$; (2) trait-dependent transmission and progression functions that link viral phenotype to drug susceptibility; and (3) continuous-time evolutionary dynamics governed by Lande’s equation. For other long-acting injectables, such as lenacapavir, which targets the HIV capsid and has a half-life of approximately six months, the same framework can be implemented by substituting drug-specific PK parameters and mechanism-dependent resistance functions. More generally, the qualitative conclusions regarding the relative importance of drug coverage breadth (σ) and initial trait configuration are expected to extend beyond CAB-LA. Any long-acting agent that generates fluctuating or declining drug concentrations between doses will produce analogous time-dependent fitness seascapes, potentially concentrating selection for resistance during periods of reduced drug exposure. Quantitative predictions, however, remain drug- and mutation-specific and therefore require empirical calibration of the relevant resistance phenotypes and PK/PD relationships.
